# Coding Synthetic Chemistry Strategies for Furan Valorization into Bacterial Designer Cells**

**DOI:** 10.1002/cssc.202201790

**Published:** 2022-12-12

**Authors:** Yu‐Chang Liu, Zhong‐Liu Wu, Jan Deska

**Affiliations:** ^1^ Department of Chemistry University of Helsinki A.I. Virtasen aukio 1 00560 Helsinki Finland; ^2^ Department of Chemistry Aalto University Kemistintie 1 02150 Espoo Finland; ^3^ CAS Key Laboratory of Environmental and Applied Microbiology Environmental Microbiology Key Laboratory of Sichuan Province Chengdu Institute of Biology Chinese Academy of Sciences Chengdu 610041 P. R. China

**Keywords:** artificial enzyme modules, biocatalysis, co-expression, platform chemicals, whole cells

## Abstract

Following a synthetic chemistry blueprint for the valorization of lignocellulosic platform chemicals, this study showcases a so far unprecedented approach to implement non‐natural enzyme modules in vivo. For the design of a novel functional whole cell tool, two purely abiotic transformations, a styrene monooxygenase‐catalyzed Achmatowicz rearrangement and an alcohol dehydrogenase‐mediated borrowing hydrogen redox isomerization, were incorporated into a recombinant bacterial host. Introducing this type of chemistry otherwise unknown in biosynthesis, the cellular factories were enabled to produce complex lactone building blocks in good yield from bio‐based furan substrates. This whole cell system streamlined the synthetic cascade, eliminated isolation and purification steps, and provided a high degree of stereoselectivity that has so far been elusive in the chemical methodology.

## Graphical abstract

**Designer cells**: A microbial factory is designed to directly convert furfuryl alcohols to synthetically valuable and enantioenriched delta‐lactones. Pairing Achmatowicz ring expansions and borrowing hydrogen redoxisomerizations, two otherwise non‐natural transformations, this study highlights the potential of introducing novel biocatalytic tools into microbes to widen the scope of bioproduction platforms.

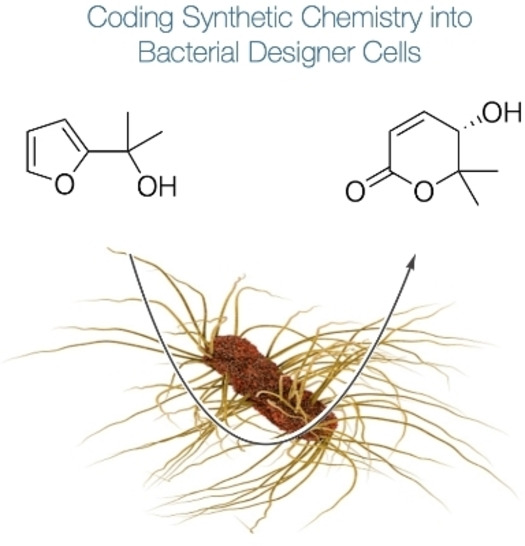

## Introduction

The systematic expansion of the reaction scope of natural systems beyond the biosynthetically encoded transformations has received considerable attention over the past decade. Here, chemically relevant key transformations are used as a blueprint for the design of new‐to‐nature enzyme catalysts. The different approaches to reach abiotic functions can range from the rational de novo design of biocatalysts,^[1]^ over directed evolution to nurture trace activities,^[2]^ all the way to repurposing of wild‐type proteins based on the exploitation of intrinsic promiscuity features^[3]^ or external triggers such as photoexcitation.^[4]^ While the reverse biomimetics approach, using chemistry as an inspirational source for the development of biological tools,^[5]^ can be seen as one of the current success stories in the field of biocatalysis, so far in vivo applications of artificial enzyme modules in genetically tailored microbes have been rare.^[6]^ More importantly, these examples were always limited to the expression of a single protein featuring the artificial function without its incorporation into an existing metabolic network that could make use of the new reactivity.

**Scheme 1:**
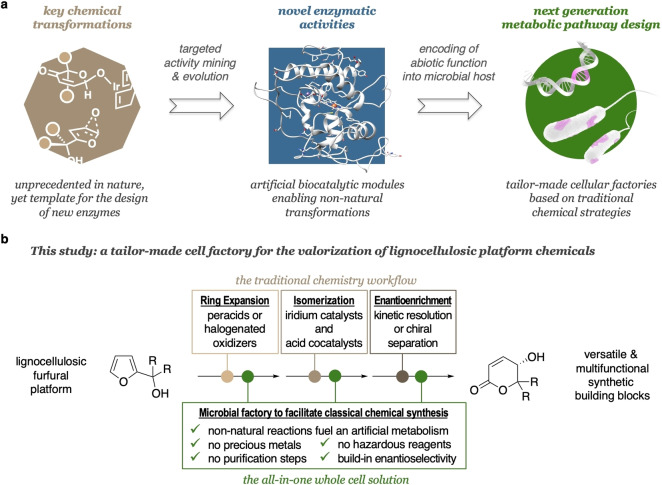
Intended workflow from a traditional chemical synthesis strategy towards chemo‐inspired tailor‐made cell factories. (a) Inspiration from chemical methodology informs the development of novel biocatalysis modules, which in turn allow to break away from the current limitations in metabolic engineering. (b) Streamlining existing valorization strategies of bio‐renewables through novel designer cells.

Currently, there seems to be a missing link between these reaction‐driven advances in biocatalysis and the obvious long‐term application, that is, to make enzymology outside the limited native biosynthetic scope part of modern metabolic engineering.^[7]^ Offering very elegant and highly streamlined solutions for the biological synthesis of complex metabolites, the development of microbial producers of valuable chemicals such as pharmaceuticals^[8]^ or polymer precursors^[9]^ has recently seen a tremendous boom, where transgenic strains combine elements of different biosynthetic pathways to assemble novel bioproduction platforms. Nevertheless, while these techniques become rapidly more elaborate and effective, the limited reaction portfolio of native biosynthesis hampers an even wider and more market‐focused implementation. Encoding abiotic functions into microbial hosts not just as a stand‐alone feature but as central element in a next‐generation pathway design appears as promising approach that allows to produce valuable chemicals through metabolic engineering and bioproduction that would not necessarily be accessible by relying on found in the realm of normal biosynthesis (Scheme 1a).

In this case study, we disclose a successful implementation of multiple non‐natural tools in a whole cell scenario, giving rise to functionalized and stereochemically defined delta‐lactones that constitute valuable structural units in bioactive compounds.^[10]^ The furfural platform is widely considered as an important pillar of bio‐renewable value chains.^[11]^ The furans as major intermediates in lignocellulosic biorefinery offer a rich reactivity for further derivatizations, and synthetic chemistry has successfully developed routes for their valorization to versatile and multifunctional building blocks.^[12]^ However, the existing chemistry is not particularly green, relies on hazardous or non‐sustainable reagents/catalysts, and suffers selectivity issues. In this work, we translate the previously reported traditional chemistry workflow into a bacterial system that interconnects native and non‐native reactivities to arrive at a strain that effectively mimics the chemical blueprint (Scheme 1b). Complementing existing bacterial furan valorizations targeting polymer building blocks,^[13]^ this report constitutes the first example of an all‐in‐one whole cell solution where a microbial factory carries a series of abiotic functions to facilitate classical chemical synthesis in a multi‐step fashion.

## Results and Discussion

The key obstacle in the development of a whole cell solution, in which non‐natural transformations ought to be utilized in

reaction cascades outside the host organism's natural metabolism, is clearly the identification of protein‐based solution for the necessary abiotic functions. Not just in the case of our furfural refinement approach, previously identified biocatalytic tools can only act as a starting point for an activity mining as whole cell cascades demand much stricter abidance to a range of parameters and properties than in vitro enzyme catalysis. Combining two non‐natural transformations, the implementation of our furfural platform valorization strategy required the identification of at least two individual enzymes with the prerequisites to (a) show high activity in the desired Achmatowicz rearrangement and the borrowing hydrogen redox isomerization, respectively, (b) operate in a nicotinamide‐dependent fashion in order to effectively couple the processes with each another, and (c) warrant the compatibility of co‐expression between multiple heterogenous genes in a single host cell, making sure each component can work in the designated pathway. Furfuryl alcohol **1 a** was chosen as model compound for the development of a whole cell furan refinement system. While traditional synthetic chemistry relies on brominating agents such as *N*‐bromosuccinimide for the ring expansion reaction (Table 1, entry 1),^[14]^ the combination of chloroperoxidase and glucose oxidase delivers an elegant biocatalytic alternative and provides comparable yields of the intermediate pyranone **2 a** (Table 1, entry 2).^[15]^ As fungal enzyme with a rich glycosylation pattern and peroxide‐dependence instead of the desired nicotinamide‐dependence, chloroperoxidase, however, does not comply well with a design based on a bacterial host organism, and hence, we investigated a small set of monooxygenases as more compatible alternatives. The introduction of the genes corresponding to cytochrome P450 (from *B. megaterium*), cytochrome P108N7 (from *R. wratislavensis*) and styrene monooxygenase (from *Pseudomonas* sp LQ26), respectively, into *E. coli* BL21(DE3) DnemA resulted in recombinant cells that were cultivated and analyzed for reactivity in the conversion of **1 a** (Table 1, entries 3–5). The DnemA strain was chosen to prevent undesired reductase activity on the enone‐type intermediates/product.^[16]^ Only whole cell catalysts carrying the flavin‐dependent styrene monooxygenase exhibited significant activity, and to our delight, the desired pyranone **2 a** could be obtained with a high selectivity of 73 %. Next, the second key transformation to the target lactone product **3 a** was addressed with a screening on potential redox isomerization biocatalysts. Originally described by Tang and co‐workers using an iridium catalyst and 2,4‐dichlorobenzoic acid as co‐activator (Table 1, entry 6),^[17]^ in 2018, we reported on the utilization of commercial alcohol dehydrogenases as proteinogenic counterpart for the borrowing hydrogen transformation of **2 a** to yield **3 a** not just in superior yields, but with very high enantiopurity of with an enantiomeric ratio (e.r.) of up to 98 : 2 (Table 1, entry 7), a feature that has so far not been achieved in this reaction by transition metal‐based catalysts.^[18]^ The same *E. coli* BL21(DE3) ΔnemA was exploited as host for the expression of five different nicotinamide‐dependent dehydrogenases (Table 1, entries 8–12). Whereas the dehydrogenation to the corresponding ketolactone **4 a** was observed as a more or less common feature, only the dehydrogenase from *Lactobacillus kefir* was able to perform the full redox isomerization (lactol dehydrogenation and ketoreduction). Here, LkADH‐expressing *E. coli* did not only convert *rac*‐**2 a** quantitatively (at up to 10 g L^−1^ within 4 h), but in an enantioconvergent manner rendered the product lactone **3 a** as single enantiomer (e.r.>99 : 1), exceeding the selectivities of any commercially available biocatalyst so far. However, to our very surprise, four out of five incubations of **2 a** with the recombinant cells actually delivered significant amounts of furfuryl alcohol **1 a**, reaching up to 30 % in case of *Chryseobacterium* ADH (Table 1, entry 11).

**Table 1:** Abiotic reactions in living organisms: Screening for whole cell solutions to mimic the existing key chemical transformations.

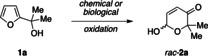				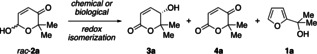						
Oxidation catalysis					Isomerization catalysis					
Entry^[a]^	Reagents/catalysts	Conv. [%]	Yield 2a [%]	Conditions	Entry^[b]^	Reagents/catalysts	Yield 3a [%]	e.r. 3a [%]	Yield 4a [%]	Yield 1a [%]
1	*N*‐bromosuccinimide, NaOAc, NaHCO_3_	>99	85	chemical	6	[Ir(cod)Cl]_2_, 2,4‐dichlorobenzoic acid	61	*rac*	0	0
2	d‐Glc, glucose oxidase (*A. niger*), chloroperoxidase (*C. fumago*)	>99	81	biocatalytic	7	NADP^+^, KRED110, alcohol dehydrogenase	83	98 : 2	5	0
3	cytochrome P450 (*Bacillus megaterium*)	<5	0	whole cell^[c]^	8	alcohol dehydrogenase (*Rhodococcus erythropolis*)	3	n.d.	85	0
				whole cell^[c]^	9	alcohol dehydrogenase (*Ogataea wickerhamii*)	0	–	51	12
4	cytochrome P108N7 (*Rhodococcus wratislaviensis*)	0	0	whole cell^[c]^	10	alcohol dehydrogenase (*Candida maris*)	0	–	47	20
				whole cell^[c]^	11	alcohol dehydrogenase (*Chryseobacterium* sp. CA49)	0	–	0	30
5	styrene monooxygenase (*Pseudomonas* sp LQ26)	>99	73	whole cell^[c]^	12	alcohol dehydrogenase (*Lactobacillus kefir* DSM20587)	97	99 : 1	0	2

[a] Conditions entries 3–5: 1 mg **1 a**, 0.1 g fresh recombinant *E. coli* cells, suspended in 1 mL phosphate buffer (0.1 m, pH 7.0) at 30 °C for 20 h. [b] Conditions entries 8–12: 1 mg *rac*‐**2 a**, 0.1 g fresh recombinant *E. coli* cells, suspending in 1 mL phosphate buffer 7.0 (0.1 m, pH 7.0) at 30 °C for 20 h. [c] *E. coli* BL21(DE3) DnemA, genes introduced via pET15 or pRSFDuet‐1.

**Scheme 2:**
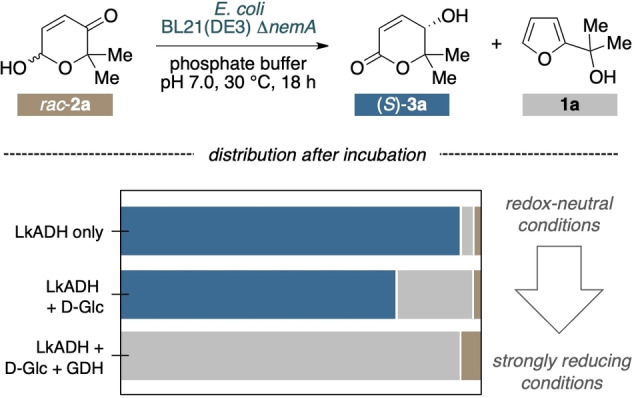
Unprecedented retro‐Achmatowicz pathway as side reaction under reductive pressure.

While the original screening to identify suitable expressible enzyme modules for the two key transformations was successful, the rather general appearance of the furfuryl alcohol **1 a**, the actual starting point of a potential synthetic cascade, with most of the tested dehydrogenases, raised questions on the validity of our design. In order to assess the risk of a non‐productive loop, perpetually converting **1 a** to **2 a** and straight back to **1 a** under the consumption of reduction equivalents, the most effective isomerase module based on *L. kefir* alcohol dehydrogenase was exposed to varying levels of reductive pressure (Scheme 2). Resting cells of the bacterial host overexpressing only the artificial isomerase in a non‐reductive medium clearly performed their designated role with virtue, yielding the optically pure lactone (*S*)‐**3 a** in 97 %, with only traces of the furfuryl alcohol **1 a**. Supplementing the medium with glucose in order to drive the endogenous production of reducing nicotinamides, furan production was slightly upregulated, yet the desired lactone remained the major metabolite. In stark contrast, introducing the glucose dehydrogenase gene alongside the artificial isomerase in a co‐expression system, the modified recombinant bacterial host also converted the pyranone **2 a** with ease, however, the selectivity switched entirely in favor of the furan product. This conversion of **2** to **1** represents a so far unprecedented direct retro‐Achmatowicz reaction, that without doubt merits further attention as a possible biocatalytic tool. In the development of an effective whole cell cascade to produce enantiomerically pure lactones, however, this parasitic retro‐Achmatowicz ring contraction poses a significant challenge demanding strict regulation of the redox balance within the cellular factory.

**Scheme 3:**
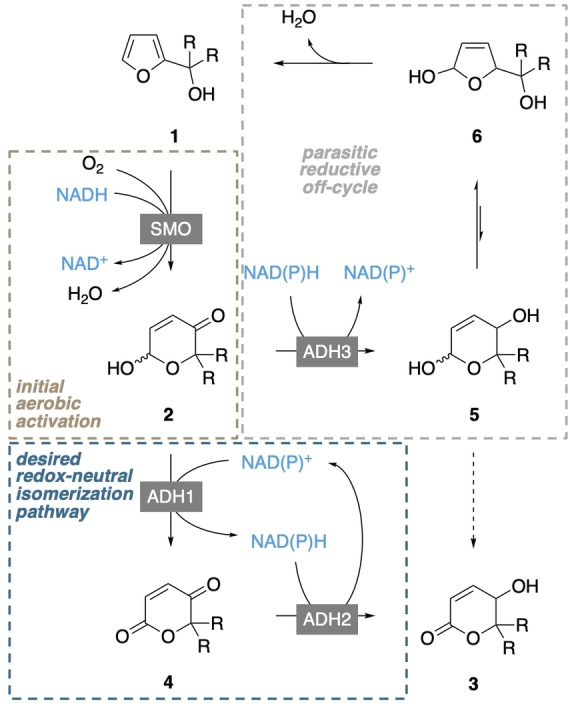
Productive and non‐productive pathways for the biotransformation of furfuryl alcohols based on a cascade design that translates the chemistry blueprint literally.

The initial investigations in the desired route to produce enantiopure lactones directly from biorefinery furans revealed an extended picture of fundamental steps and dependencies (Scheme 3), and a number of possible points of intervention to regulate the biosynthetic machinery towards the chiral heterocyclic products. In an initial ring expansion, a monooxygenase converts the biogenic furans **1** to the intermediary pyranones **2** with consumption of dioxygen and reduced nicotinamides. From here, a catalytic bifurcation emerges, where the productive or non‐productive outcome is decided by the selectivity to either reductively or oxidatively activate **2**. The desired route (isomerase‐mode) proceeds with the oxidation of the hemiacetal moiety through an alcohol dehydrogenase utilizing NAD(P)^+^ to form the ketolactones **4** alongside with NAD(P)H. With the same (or a second, complementary) dehydrogenase the ketone is finally reduced to give rise to the hydroxylactones **3** and recycle NAD(P)^+^ to drive the redox‐self‐sufficient isomerization. If oxidation of **2** remains slow, the risk for a reductive parasitic off‐cycle pathway is increased in which the alcohol dehydrogenase consumes NAD(P)H to deliver the reduced six‐membered hydroxylactol **5**. After ring contraction to the corresponding five‐membered lactol **6**, spontaneous dehydration closes the non‐productive cycle and delivers the furanoic starting material, after net consumption of two equivalents of reducing nicotinamide. There would apparently also be a pathway plausible to access the desired delta‐lactone **3** from the hydroxy hemiacetal **5** through another ADH‐mediated dehydrogenation under consumption of NAD(P)^+^. In practice however, any attempts (also in vitro through the forced reduction of **2** by ADH) have so far led only to five‐membered products, indicating that the equilibrium between the hemiacetal rapidly removes any six‐membered **5** and thus prevents this alternative route. Both the selectivity of the alcohol dehydrogenase(s) as well as the balance between oxidizing and reducing cofactors in the bacterial designer cells will thus influence this delicate network of reactions. Especially the ambiguity regarding reducing cofactors, being crucial to catalyze the furan oxygenation while also offering an entry into the undesired off‐cycle need to be carefully addressed in the actual cascade design.

The implementation of redox networks in *E. coli* merging monooxygenase and dehydrogenase activities has been successfully employed in a number of microbial factories, and numerous co‐expression strategies have been discussed in literature.^[19]^ In a first attempt to assemble a suitable cellular factory, the coding sequences of styrene monooxygenase and LkADH were amplified and engineered into the commercially available pRSFDuet‐1^T^ vector, giving two co‐expression plasmids SMO‐LK and LK‐SMO under the control of two T7 promoters. The two plasmids were transformed into *E. coli* Δ*nemA* to produce *E.coli* (SMO‐LK) and *E. coli* (LK‐SMO), respectively. The strains were cultivated in LB medium with kanamycin, the target proteins were successfully expressed, and the fresh resting cells were employed to transform model substrate **1 a**. Interestingly, the two different architectures exhibited an orthogonal selectivity profile. Whereas *E. coli* (LK‐SMO) only performed the initial furan oxidation with no traces of the desired lactone (Table 2, entry 2), *E. coli* (SMO‐LK) managed to act as bacterial producer for the final product (*S*)‐**3 a** with an optical purity of 99 % (Table 2, entry 3). The excessive amount of remaining furan substrate left in either of the reaction mixtures, however, is indicative of an inefficient supply of reduced nicotinamide, and perhaps also a result of issues related to the co‐expression of Lk‐ADH and SMO from the same plasmid. Considering the highly oxidative capability of SMO on **1 a** as described above, we decided to ameliorate nicotinamide regeneration in order to support the SMO‐catalyzed oxidation by means of another dehydrogenase in the single whole cell system. SMO is a strictly NADH‐dependent monooxygenase whilst Lk‐ADH is preferentially using NADPH, allowing to selectively address only one of the key oxidoreductases. The coding sequence of formate dehydrogenase (FDH) was engineered into the pET‐15b vector and transformed into *E. coli* BL21(DE3) Δ*nemA* together with the pRSFD‐SMO‐LK plasmid yielding *E. coli* (SMO‐LK/FDH). Unfortunately, this construct proved to be ineffective as no conversion of furan **1 a** was detected even after an extended incubation period (Table 2, entry 4). It appears somewhat surprising that not even the mediocre conversions of *E*. coli (SMO‐LK) could be matched, as systems combining pRSFDuet and pET‐15b represent rather typical designs for the co‐expression of multiple proteins, including combinations of alcohol dehydrogenases and flavin‐dependent monooxygenases.^[20]^ Likely, formate as strong reductant may play a role in interfering with the redox cascade similar to the glucose dehydrogenase. The previously elaborated reaction network, however, also offers an alternative way to supply reduced nicotinamides for the SMO‐mediated Achmatowicz oxygenation. As discovered in the initial screening, certain alcohol dehydrogenases do indeed engage in the dehydrogenation of **2** and provide ketolactone **4**, although they are incapable to conduct the complete redox‐isomerization. Introduction of one of those biocatalytic tools would not only strongly influence the catalytic bifurcation in favor of the desired oxidative activation, but it would also create a redox‐self‐sufficient unit to push the so far ineffective furan rearrangement by improved supply of NADH fueling the SMO module. Therefore, alcohol dehydrogenase ReADH with its NADH cofactor preference (and also its low ketoreductase activity in the reduction of **2** or **4**) was chosen as a supplementary module alongside SMO and LkADH in vivo. To test our hypothesis, a binary co‐expression system combining ReADH and SMO was assembled and in fact delivered almost exclusively the ketolactone **4 a** (Table 2, entry 5). In order to optimize the expression level of the three genes required for the complete cascade, the coding sequences of SMO, LKADH, and READH were engineered on pRSFDuet‐1^T^ and pET‐15b to give a total of four different expression cassettes, namely, **SLR1** (pRFDuet‐SMO‐LK/pET15b‐RE), **SLR2** (pRFDuet‐LK‐SMO/pET15b‐RE), **SLR3** (pRFDuet‐RE‐LK/pET15b‐SMO), **SLR4** (pRFDuet‐Re‐SMO/pET15b‐LK). Each operon was under the control of individual T7 promotors. Similar vector combinations were widely studied, and the choice of cassette for the individual genes allows to modulate protein expression levels without extensive manipulation in the plasmid through different promoters or terminators. As shown by Zhang and co‐workers for a given combination of target genes, expression from pET plasmids offers higher higher protein levels compared to Duet‐type plasmids, and within a two‐polylinker plasmid like pRSFDuet, the second MCS should likely appear as the stronger position.^[21]^ Nevertheless, with SMO and LkADH as critical tools and ReADH as booster, and some reactions being irreversible while others may show reversibility, we opted for a simple combinatorial approach to construct four different triple expression systems and evaluate their performance based on effective conversion rather than individual protein expression levels. The four sets of plasmids were transformed into *E. coli* BL21(DE3) Δ*nemA*, respectively, and the whole cell catalysts were prepared as described before. The four strains were applied to the biotransformation of **1 a** and while all exhibited some activity to produce the lactone (*S*)‐**3 a** (Table 2, entries 6–9), SLR4 showed the best performance without the accumulation of any intermediates. Even though the first two steps in the design include SMO and ReADH as redox‐self‐sufficient biocatalytic entity,^[22]^ the overall process requires external reductants. Thus, utilization of the commonly employed cofactor recycling using isopropanol to drive dehydrogenase processes appeared as a logical measure to overcome the mediocre yields of **3 a** (max. 32 %). Gratifyingly, supplementing the medium with 0.2 % (*v*/*v*) isopropanol resulted in a highly effective cellular factory providing not only full conversion but 91 % selectivity for the enantiopure lactone (*S*)‐**3 a** (Table 2, entry 10). Excessive supply of isopropanol on the other hand led to an apparent inhibition, most likely by deviating the artificial metabolism towards the non‐productive off‐cycle (Table 2, entry 11).

**Table 2:** Co‐expression systems targeting the full implementation of the abiotic cascade in a recombinant *E. coli* host.

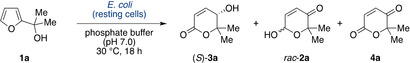						
Entry	Plasmid(s) inserted	Additive [%]	Conv. [%]	Yield 3a [%]	Yield 2a [%]	Yield 4a [%]
1	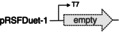	none	<5	0	0	0
2	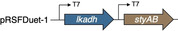	none	14	0	13	2
3	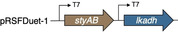	none	12	11	0	0
4	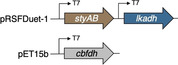	NaHCO_2_ (5 mm)	<5	0	0	0
5	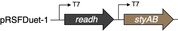	none	40	0	4	36
6	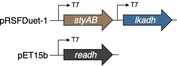	none	3	2	0	0
	SLR1					
7	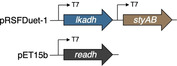	none	12	12	0	0
	SLR2					
8	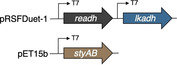	none	8	7	0	0
	SLR3					
9	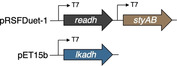	none	37	32	0	0
10	SLR4	*i*PrOH (0.2 vol%)	>99	91	0	0
11		*i*PrOH (2 vol%)	16	13	0	0

**Figure 1:**
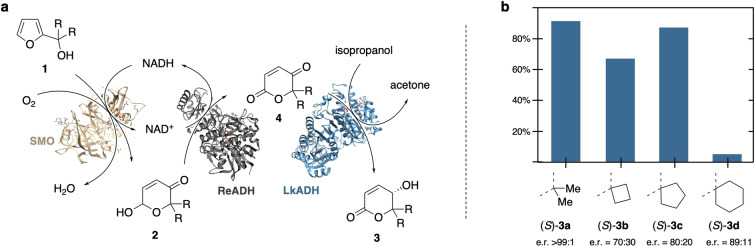
Production of enantioenriched hydroxylactones in a tailor‐made *E. coli* strain. (a) Revised biocatalytic network enabling a direct conversion of furfuryl alcohols. (b) Substrate scope of preparative scale whole cell biotransformations.

Taking into account the apparent side reaction profile and the individual characteristics of the three enzyme modules, the most likely cascade network was concluded to follow the sequence depicted in Figure 1a. In contrast to the original design (Scheme 2), our most effective cellular factory deviates from the initial draft in that the redox‐self‐sufficient isomerase module (i. e. net‐neutral in NAD(P)H with one single dehydrogenase biocatalyst) has been replaced by a net‐NADH‐neutral conversion of the furfuryl alcohols (**1**) to the ketolactone intermediates (**4**) by cooperative action of styrene monooxygenase and ReADH, while the net‐reducing event has been relocated towards the end of the sequence. With this ternary expression system in place, a powerful whole cell assembly for the direct production of enantiomerically pure delta‐lactones from basic wood‐derived furan precursors was obtained. To our delight, the bacterial construct was not limited to the model furan **1 a** but accepted also structural variations (Figure 1b). Incubation with 1‐furylcycloalkanols resulted in the production of the synthetically highly interesting spirolactones (*S*)‐**3 b** and (*S*)‐**3 c** in good yields. The current limitation of this initial cellular factory design appears to lie in the substrate lipophilicity and or sterics as cycloalkanols, as the spirocyclic products did not yet reach the same high optical purities, and as cyclohexanol **1 d** was only poorly converted by the same microbial system. Future investigations on the herein presented design will therefore target the influence of cosolvents and emulsifiers as well as controlling fluxes that may help to expand the substrate scope in order to make full use of this exciting new biological tool for the valorization of simple lignocellulosic platform chemicals to stereochemically defined building blocks for fine chemicals, pharmaceuticals, and fragrances.

## Conclusion

We were able to successfully incorporate a truly non‐natural reaction cascade into a simple bacterial host, and to fine tune the co‐expression system by rather simple means to act as highly selective producer of multifunctional synthetic building blocks from a basic lignocellulose‐derived feed. The thus obtained bioproduction platform exhibits a range of benefits over its organic‐synthetic template, as the valorization of biorefinery furan in the designer cells proceeds without any intermediate isolation or purification steps, rendering analytically pure lactones after a simple extraction in very high overall yields. Avoiding any kind of hazardous or precious metal‐based additives, the biological interpretation of this synthetic chemistry strategy appears not just much more sustainable than the traditional blueprint but also benefits from the intrinsic nature of the key enzyme functions in that the products are obtained with good to excellent enantioselectivity. In addition, the same study provided also whole new insights into the established underlying chemistry of the target process, where the discovery of a so far unprecedented retro‐Achmatowicz reaction will unequivocally serve as starting point for mechanistic investigations on this transformation.

## Experimental Section

### General remarks

*E.coli* BL21(DE3) and *E.coli* Top10 competent cells, restriction enzymes, high‐fidelity DNA polymerase, and T4 ligase were purchased from Thermo Fisher. The other molecular biological materials were gifted by Prof. Zhong‐Liu Wu, Chengdu Institute of Biology, Chinese Academy of Science, including plasmids encoding alcohol dehydrogenase: pET‐15b‐*Re*ADH from *Rhodococcus erythropolis* (GenBank accession No. AY161280), pET‐15b‐*Lk*ADH from *Lactobacillus kefir* (GenBank accession No. AY267012), pRSFD‐*Cm*ADH from *Candida maris* (GenBank accession No. BAK26806), pRSFD‐*Ow*ADH from *Ogataea wickerhamii* (GenBank accession No. BAF36551), and pET‐28a‐*Ch*KRED20 from *Chryseobacterium* sp. CA49 (GenBank accession No. KC342005), as well as plasmids encoding monoxygenase pET‐28a‐*Sty*AB from *Pseudomonas* sp. LQ26 (GenBank accession No. GU593979), pET‐28a‐CYP_BM3_ from *Bacillus megaterium*, and pET‐28a‐CYP_108N7_ from *Rhodococcus wratislaviensis* (GenBank accession No. WP_037231399), plasmid pET‐28a‐FDH encoding formate dehydrogenase from *Candida boidinii*, vectors pET‐15b and pRSFDuet‐1^T^, and host cells *E.coli* BL21(DE3) Δ*nemA*. All synthetic reactions that were carried out under argon atmosphere were performed with dry solvents using anhydrous conditions. Dry solvents were taken from a solvent drying system MB‐SPS‐800 from M‐Braun. Commercially available reagents were used without further purification. Enzymatic reactions were performed under non‐inert conditions on an orbital shaker in caped glass vials. Column chromatography was performed with silica gel from Merck (Millipore 60, 40–60 μm, 240–400 mesh). Reactions were monitored by thin layer chromatography (TLC) carried out on Machery‐Nagel precoated silica gel plates (TLC Silica gel 60 F_254_). Visualisation of the TLC plates was done by using UV light and staining with a basic potassium permanganate solution. ^1^H and ^13^C nuclear magnetic resonance (NMR) spectra were recorded on a Bruker AV‐400 instrument at 20 °C. Chemical shifts are reported in parts per million (ppm) calibrated using residual non‐deuterated solvents as internal reference [CHCl_3_ at *δ*=7.26 ppm (^1^H NMR) and 77.2 ppm (^13^C NMR). Infrared (IR) spectra were recorded on a Bruker ALPHA Eco‐ATR spectrometer, absorption bands are reported in wavenumbers [cm^−1^]. Gas chromatography (GC) was performed on a Hewlett Packard HP 6890 Series GC System using a Macherey‐Nagel FS‐Lipodex A and Macherey‐Nagel FS‐Lipodex E column (25 m×0.25 mm), helium, 1.0 mL min^−1^; temperature program: 50 °C (1 min)/5 °C min^−1^ (35 min)/120 °C (15 min). High‐performance liquid chromatography (HPLC) analysis was performed on an Agilent 1100 system with a G1312 A binary pump and a G1312B diode array detector using analytical Daicel Chiralpak column (250 mm×4.6 mm; ADH or AS−H).

### Construction of co‐expression plasmids

The DNA fragment encoding Styrene monooxygenase was amplified from vector pET‐28a‐StyAB. The DNA fragment encoding alcohol dehydrogenase READH or LKADH was amplified from vector pET‐15b‐*Re*ADH and pET‐15b‐*Lk*ADH, separately. The DNA fragments encoding formate dehydrogenase was amplified from pET‐28a‐FDH. The PCR products were purified, double digested with the corresponding restriction enzymes, and ligated into the corresponding cassette of pRFDuet‐1^T^ or pET‐15b (see Supporting Table S1). The ligation mixture was transformed into *E. coli* Top10 competent cells. Single colonies were selected on LB agar plate supplemented with kanamycin (50 μg mL^−1^) or ampicillin (50 μg mL^−1^). The expression plasmid was identified by sequencing the ORF regions of the target DNA.

### Representative procedure for the co‐expression of SMO, ReADH, and LkADH

Recombinant *E. coli* BL21(DE3)Δ*nemA* harboring pRSFD‐ReADH/SMO and pET‐15b‐LkADH were cultivated overnight at 37 °C in Luria‐Bertani (LB) medium containing kanamycin (50 μg mL^−1^) and ampicillin (50 μg mL^−1^), or only kanamycin (50 μg mL^−1^). Then 1 mL of overnight culture was inoculated into 100 mL of LB containing kanamycin (50 μg mL^−1^) and ampicillin (50 μg mL^−1^), or only kanamycin (50 μg mL^−1^), and then incubated at 37 °C for 2 h. The expression was induced by the addition of 0.1 mm isopropyl‐β‐d‐thiogalactopyranoside (IPTG), and the incubation was continued for 15 h at 20 °C with rotary shaking at 200 rpm. The cells were harvested by centrifugation at 5000 rpm and washed twice with potassium phosphate buffer (0.1 m, pH 7.0).

### Representative procedure for the direct conversion of furans to lactones

**(*S*)‐5‐Hydroxy‐6,6‐dimethyl‐5,6‐dihydro‐2*H*‐pyran‐2‐one (3 a)**: **1 a** (10 mg, 79.3 μmol) was dissolved in 1 mL of potassium phosphate buffer (0.1 m, pH 7.0) containing fresh recombinant *E. coli* cells (pRFDuet‐Re‐SMO/pET15b‐LK; 1.0 g) and isopropanol (20 μL) The mixture was incubated at 30 °C with shaking at 200 rpm for 20 h. The reaction was terminated by extraction with ethyl acetate. The combined organic layer was dried over anhydrous Na_2_SO_4_ and concentrated under reduced pressure. The residue was purified by column chromatography (SiO_2_, hexane/ethyl acetate, 1 : 1) yielding (*S*)‐**3 a** (10.3 mg, 72.4 μmol, 91 %, 99 % *ee*) as colorless oil. *R*_f_=0.34 (hexane/ethyl acetate 1 : 1). ^1^H NMR (400 MHz, CDCl_3_): *δ*=6.81 (dd, *J*=9.8 Hz, *J*=4.0 Hz, 1H), 6.05 (dd, *J*=9.8 Hz, *J*=1.3 Hz, 1H), 4.20 (s, 1H), 2.15 (br, 1H), 1.46 ppm (s, 6H). ^13^C NMR (100 MHz, CDCl_3_): *δ*=162.9, 145.4, 121.6, 83.4, 68.6, 26.4, 21.8 ppm. HPLC (*Chiralpak AS*, hexane/isopropanol 9 : 1, 0.8 mL min^−1^, 210 nm): *t*_R_ [(*S*)‐**2 f**]=34.2 min, *t*_R_ [(*R*)‐**2 f**]=37.5 min (e.r.>99 : 1).

## Conflict of interest

The authors declare no conflict of interest.

1

## Supporting information

As a service to our authors and readers, this journal provides supporting information supplied by the authors. Such materials are peer reviewed and may be re‐organized for online delivery, but are not copy‐edited or typeset. Technical support issues arising from supporting information (other than missing files) should be addressed to the authors.

Supporting Information

Supporting Information

Supporting Information

Supporting Information

## Data Availability

The data that support the findings of this study are available in the supplementary material of this article.

## References

[1a] J. B. Siegel , A. Zhanghellini , H. M. Lovick , G. Kiss , A. R. Lambert , J. L. St. Clair , J. L. Gallaher , D. Hilvert , M. H. Gelb , B. L. Stoddard , K. N. Houk , F. E. Michael , D. Baker , Science 2010, 329, 309–313; 20647463 10.1126/science.1190239 PMC3241958

[1b] R. Blomberg , H. Kries , D. M. Pinkas , P. R. E. Mittl , M. G. Grütter , H. K. Privett , S. L. Mayo , D. Hilvert , Nature 2013, 503, 418–421. 24132235 10.1038/nature12623

[2a] P. S. Coelho , E. M. Brustad , A. Kannan , F. H. Arnold , Science 2013, 339, 307–310; 23258409 10.1126/science.1231434

[2b] V. Tyagi , G. Sreenilayam , P. Bajaj , A. Tinoco , R. Fasan , Angew. Chem. Int. Ed. 2016, 55, 13562–13566; 10.1002/anie.201607278 PMC5189672 27647732

[2c] C. K. Prier , R. K. Zhang , A. R. Buller , S. Brinkmann-Chen , F. H. Arnold , Nat. Chem. 2017, 9, 629–634; 28644476 10.1038/nchem.2783 PMC5555633

[2d] R. K. Zhang , K. Chen , X. Huang , L. Wohlschläger , H. Renata , F. H. Arnold , Nature 2019, 565, 67–72; 30568304 10.1038/s41586-018-0808-5 PMC6440214

[2e] N. W. Goldberg , A. M. Knight , R. K. Zhang , F. H. Arnold , J. Am. Chem. Soc. 2019, 141, 19585–19588. 31790588 10.1021/jacs.9b11608 PMC6924163

[3a] C. Jäger , J. Deska , ChemRxiv. 2021, 10.26434/chemrxiv.14562048;

[3b] D. Thiel , F. Blume , C. Jäger , J. Deska , Eur. J. Org. Chem. 2018, 20, 2717–2725;

[3c] J. Naapuri , J. D. Rolfes , J. Keil , C. Manzuna Sapu , J. Deska , Green Chem. 2017, 19, 447–452.

[4] L. Schmermund , V. Jurkaš , F. F. Özgen , G. D. Barone , H. C. Büchsenschütz , C. K. Winkler , S. Schmidt , R. Kourist , W. Kroutil , ACS Catal. 2019, 9, 4115–4144.

[5a] N. J. Turner , E. O′Reilly , Nat. Chem. Biol. 2013, 9, 285–288; 23594772 10.1038/nchembio.1235

[5b] W. Finnigan , L. J. Hepworth , S. L. Flitsch , N. J. Turner , Nat. Catal. 2021, 4, 98–104. 33604511 10.1038/s41929-020-00556-z PMC7116764

[6a] P. S. Coelho , Z. J. Wang , M. E. Ener , S. A. Baril , A. Kannan , F. H. Arnold , E. M. Brustad , Nat. Chem. Biol. 2013, 9, 485–487; 23792734 10.1038/nchembio.1278 PMC3720782

[6b] S. B. J. Kan , R. D. Lewis , K. Chen , F. H. Arnold , Science 2016, 354, 1048–1051. 27885032 10.1126/science.aah6219 PMC5243118

[7a] S. P. France , L. J. Hepworth , N. Turner , S. L. Flitsch , ACS Catal. 2017, 7, 710–724;

[7b] K. B. Otte , B. Hauer , Curr. Opin. Biotechnol. 2015, 35, 16–22. 25585003 10.1016/j.copbio.2014.12.011

[8a] D.-K. Ro , E. M. Paradise , M. Ouellet , K. J. Fisher , K. L. Newman , J. M. Ndungu , K. A. Ho , R. A. Eachus , T. S. Ham , J. Kirby , M. C. Y. Chang , S. T. Withers , Y. Shiba , R. Sarpong , J. D. Keasling , Nature 2006, 440, 940–943; 16612385 10.1038/nature04640

[8b] X. Luo , M. A. Reiter , L. d'Espaux , J. Wong , C. M. Denby , A. Lechner , Y. Zhang , A. T. Grzybowski , S. Harth , W. Lin , H. Lee , C. Yu , J. Shin , K. Deng , V. T. Benites , G. Wang , E. E. K. Baidoo , Y. Chen , I. Dev , C. J. Petzold , J. D. Keasling , Nature 2019, 567, 123–126. 30814733 10.1038/s41586-019-0978-9

[9] N. Oberleitner , A. K. Ressmann , K. Bica , P. Gärtner , M. W. Fraaje , U. W. Bornscheuer , F. Rudroff , M. D. Mihovilovic , Green Chem. 2017, 19, 267–271.

[10] S. K. Sartoria , M. Alves Nogueira Diaz , G. Diaz-Muñoz , Tetrahedron 2021, 84, 132001.

[11] R. Mariscal , P. Maireles-Torres , M. Ojeda , I. Sádaba , M. López Granados , Energy Environ. Sci. 2016, 9, 1144–1189.

[12a] A. K. Ghosh , M. Brindisi , RSC Adv. 2016, 6, 111564–111598; 28944049 10.1039/C6RA22611F PMC5603243

[12b] C. Verrier , S. Moebs-Sanchez , Y. Queneau , F. Popowycz , Org. Biomol. Chem. 2018, 16, 676–687; 29362755 10.1039/c7ob02962d

[12c] N. Li , M.-H. Zong , ACS Catal. 2021, 12, 10080–10114.

[13] X. Wang , X.-Y. Zhang , M.-H. Zong , N. Li , ACS Sustainable Chem. Eng. 2020, 8, 4341–4345.

[14] J. Deska , D. Thiel , E. Gianolio , Synthesis 2015, 47, 3435–3450.

[15a] D. Thiel , D. Doknić , J. Deska , Nat. Commun. 2014, 5, 5278; 25335580 10.1038/ncomms6278

[15b] D. Thiel , F. Blume , C. Jäger , J. Deska , Eur. J. Org. Chem. 2018, 20, 2717–2725.

[16] K. Miura , Y. Tomioka , H. Suzuki , M. Yonezawa , T. Hishinuma , M. Mizugaki , Biol. Pharm. Bull. 1997, 20, 110–112. 9013822 10.1248/bpb.20.110

[17] H.-Y. Wang , K. Yang , S. R. Bennett , S.-R. Guo , W. Tang , Angew. Chem. Int. Ed. 2015, 54, 8756–8759; 10.1002/anie.201503151 26033736

[18] Y.-C. Liu , C. Merten , J. Deska , Angew. Chem. Int. Ed. 2018, 57, 12151–12156; 10.1002/anie.201804911 PMC6468324 29984878

[19a] C. A. Müller , A. Dennig , T. Welters , T. Winkler , A. J. Ruff , W. Hummel , H. Gröger , U. Schwaneberg , J. Biotechnol. 2014, 191, 196–204; 24925696 10.1016/j.jbiotec.2014.06.001

[19b] H.-Y. Jang , E.-Y. Jeon , A.-H. Baek , S.-M. Lee , J.-B. Park , Proc. Biochem. 2014, 49, 617–622.

[20] S. Ménil , J.-L. Petit , E. Courvoisier-Dezord , A. Debard , V. Pellouin , T. Reignier , M. Sergant , V. Deyris , K. Duquesne , V. de Berardinis , V. Alphand , Biotechnol. Bioeng. 2019, 116, 2852–2863. 31389000 10.1002/bit.27133

[21] H. Chen , R. Huang , Y.-H. P. Zhang , Appl. Microbiol. Biotechnol. 2017, 101, 4481–4493. 28251267 10.1007/s00253-017-8206-8

[22] Y.-C. Liu , Z.-L. Wu , Chem. Commun. 2016, 52, 1158–1161. 10.1039/c5cc07548c 26596424

