# Supplementary material for: Coding Synthetic Chemistry Strategies for Furan Valorization into Bacterial Designer Cells
Source: ChemSusChem. 2022 Dec 12;16(2):e202201790. doi: 10.1002/cssc.202201790 (PMC10107124; doi:10.1002/cssc.202201790)

ChemSusChem

Supporting Information

Coding Synthetic Chemistry Strategies for Furan Valorization into Bacterial Designer Cells**

Yu-Chang Liu, Zhong-Liu Wu, and Jan Deska*© 2022 The Authors. ChemSusChem published by Wiley-VCH GmbH. This is an open access article under the terms of the Creative Commons Attribution License, which permits use, distribution and reproduction in any medium, provided the original work is properly cited.

Table of contents

General remarks 2

Expression of MOs and ADHs 2

Plasmid construction 3

Multi-enzyme co-expression 4

In vivo biotransformations 4

Procedures & analytical data: lactones 6

starting materials 7

NMR spectra of the compounds 9

GC traces 12

HPLC traces 19

General Remarks

E.coli BL21(DE3) and *E.coli* Top10 competent cells, restriction enzymes, high-fidelity DNA polymerase and T4 ligase were purchased from Thermo Fisher. The other molecular biological marterials were gifted by Prof. Zhong-Liu Wu, Chengdu Institute of Biology, Chinese Academy of Science, including plasmids encoding alcohol dehydrogenase: pET-15b-*Re*ADH from *Rhodococcus erythropolis* (GenBank accession No. AY161280), pET-15b-*Lk*ADH from *Lactobacillus kefir* (GenBank accession No. AY267012), pRSFD-*Cm*ADH from *Candida maris* (GenBank accession No. BAK26806), pRSFD-*Pf*ADH from *Ogataea wickerhamii* (GenBank accession No. BAF36551), and pET-28a-*Ch*KRED20 from *Chryseobacterium* sp. CA49 (GenBank accession No. KC342005), as well as plasmids encoding monoxygenase pET-28a-*Sty*AB from *Pseudomonas* sp. LQ26 (GenBank accession No. GU593979), pET-28a-CYP_{BM3} from *Bacillus megaterium*, and pET-28a-CYP_{108N7} from *Rhodococcus wratislaviensis* (GenBank accession No. WP_037231399), plasmid pET-28a-FDH encoding formate dehydrogenase from *Candida boidinii*, vectors pET-15b and pRSFDuet-1^{T}, and host cells *E.coli* BL21(DE3) Δ*nemA*.

All synthetic reactions that were carried out under argon atmosphere, were performed with dry solvents using anhydrous conditions. Dry solvents were taken from a solvent drying system MB-SPS-800 from M-Braun. Commercially available reagents were used without further purification. Enzymatic reactions were performed under non-inert conditions on an orbital shaker in caped glass vials.

Column chromatography was performed with silica gel from Merck (Millipore 60, 40-60 µm, 240-400 mesh). Reactions were monitored by thin layer chromatograhy (TLC) carried out on Machery-Nagel precoated silica gel plates (TLC Silica gel 60 F_{254}). Visualisation of the TLC plates was done by using UV light and staining with a basic potassium permanganat solution. ^{1}H- and ^{13}C-NMR-spectra were recorded on a Bruker AV-400 instrument at 20 °C. Chemical shifts are reported in parts per million (ppm) calibrated using residual non-deuterated solvents as internal reference [$CHCl_3$ at δ = 7.26 ppm (^{1}H NMR) and 77.2 ppm (^{13}C NMR). Infrared spectra were recorded on a Bruker ALPHA Eco-ATR spectrometer, absorption bands are reported in wave numbers [cm^{-1}]. Gas chromatography was performed on a Hewlett Packard HP 6890 Series GC System using a Macherey-Nagel FS-Lipodex A and Macherey-Nagel FS-Lipodex E column (25 m x 0.25 mm), helium, 1.0 ml/min; temperature program: 50 °C (1 min) / 5 °C·min^{-1} (35 min) / 120 °C (15 min). High performance liquid chromatography analysis on an Agilent 1100 system with a G1312A binary pump and a G1312B diode array detector using analytical Daicel Chiralpak column (250 mm x 4.6 mm; ADH or AS-H).

Expression of monooxygenases

Recombinant *E. coli* BL21 (DE3) Δ*nemA* harboring a plasmid of pET28a-StyAB, pET28a-BM3, or pET28a-108N7 was cultivated overnight at 37 °C in Luria–Bertani (LB) medium containing kanamycin (50 µg/ml). 1 ml of overnight culture was inoculated into 100 ml of LB containing

kanamycin (50 μg/ml), and incubated at 37 °C for 2h. The expression was induced by the addition of 0.1 mM isopropyl-β-D-thiogalactopyranoside (IPTG), and the incubation was continued for 15 h at 20 °C with rotary shaking at 200rpm. The cells were harvested by centrifugation at 5000 rpm, and washed twice with potassium phosphate buffer (0.1 M, pH 7.0).

Expression of alcohol dehydrogenase

Recombinant *E. coli* BL21 (DE3) or *E. coli* BL21 (DE3) Δ*nemA* harboring a plasmid of pET15b-*Re*ADH, pET15b-*Lk*ADH, pRSFD-*Cm*ADH, pRSFD-*Pf*ADH, or pET28a-*Ch*KRED20 was cultivated overnight at 37 °C in Luria-Bertani (LB) medium containing kanamycin (50 μg/ml) or ampicillin (50μ g/ml). 1 ml of overnight culture was inoculated into 100 ml of LB containing kanamycin (50 μg/ml) or ampicillin (50μ g/ml), and incubated at 37 °C for 6h. The expression was induced by the addition of 0.1 mM iso-propyl-β-D-thiogalactopyranoside (IPTG), and the incubation was continued for 15 h at 30 °C with rotary shaking at 200 rpm. The cells were harvested by centrifugation at 5000 rpm, and washed twice with potassium phosphate buffer (0.1 M, pH 7.0).

Construction of co-expression plasmids

The DNA fragment encoding Styrene monooxygenase was amplified from vector pET-28a-StyAB. The DNA fragment encoding alcohol dehydrogenase READH or LKADH was amplified from vector pET-15b-*Re*ADH and pET-15b-*Lk*ADH, separately. The DNA fragments encoding formate dehydrogenase was amplified from pET-28a-FDH. The PCR products were purified, double digested with the corresponding restriction enzymes, and ligated into the corresponding cassette of pRFDuet-1^{T} or pET-15b (Table S1).

The ligation mixture was transformed into *E. coli* Top10 competent cells. Single colonies were selected on LB agar plate supplemented with kanamycin (50μg /ml) or ampicillin (50 μg/ml). The expression plasmid was identified by sequencing the ORF regions of the target DNA.

Supplementary Table S1

Plasmid	Primers	Oligouncleotide sequences (5' to 3')
pRSFD-LkADH/SMO	*BamH* I_ *Lk*ADH	CCGGAATTCGATGACTGATCGTTTAAAAGGCAAAG
	Hind III_*Lk*ADH	CCCAAGCTTATTGAGCAGTGTATCCACCATCG
	Nde I_SMO	CTACATATGAAAAAGCGTATCGGTATTGTTG
	Xho I_SMO	CCGCTCGAGTTAATTCAGGGGCAGCGGATTG
pRSFD-SMO/LkADH	*BamH* I_SMO	CCGGAATTCGATGAAAAAGCGTATCGGTATTGTTG
	Hind III_SMO	CCCAAGCTTAATTCAGGGGCAGCGGATTG
	Nde I_*Lk*ADH	CTACATATGACTGATCGTTTAAAAGGCAAAG
	Xho I_*Lk*ADH	CCGCTCGAGTTATTGAGCAGTGTATCCACCATCG
pRSFD-ReADH/SMO	*BamH* I_*Re*ADH	CCGGAATTCGATGAAGGCAATCCAGTACACGAG
	Hind III_*Re*ADH	CCCAAGCTTCTACAGACCAGGGACCACAAC
	Nde I_SMO	CTACATATGAAAAAGCGTATCGGTATTGTTG
	Xho I_SMO	CCGCTCGAGTTAATTCAGGGGCAGCGGATTG
pRSFD-ReADH/LkADH	*BamH* I_*Re*ADH	CCGGAATTCGATGAAGGCAATCCAGTACACGAG
	Hind III_*Re*ADH	CCCAAGCTTCTACAGACCAGGGACCACAAC
	Nde I_*Lk*ADH	CTACATATGACTGATCGTTTAAAAGGCAAAG
	Xho I_*Lk*ADH	CCGCTCGAGTTATTGAGCAGTGTATCCACCATCG
pET15b-SMO	*Nde* I_SMO	CTACATATGAAAAAGCGTATCGGTATTGTTG
	Xho I_SMO	CCGCTCGAGTTAATTCAGGGGCAGCGGATTG
pET15b-FDH	*Nde* I_FDH	CTACATATGATGAAGATCGTTTTAGTCTTATATG
	Xho I_FDH	CCGCTCGAGTTATTTCTTATCGTGTTTACCGTAC

Co-expression of SMO and ADHs

Recombinant *E. coli* BL21(DE3)Δ*nemA* harboring a single or two vectors (as listed in Table 2) were cultivated overnight at 37 °C in Luria-Bertani (LB) medium containing kanamycin (50µg/ml) and ampicillin (50µ g/ml), or only kanamycin (50µg/ml). Then 1 ml of overnight culture was inoculated into 100 ml of LB containing kanamycin (50µg/ml) and ampicillin (50µg/ml), or only kanamycin (50µg/ml), and then incubated at 37 °C for 2 h. The expression was induced by the addition of 0.1 mM isopropyl-β-D-thiogalactopyranoside (IPTG), and the incubation was continued for 15 h at 20 °C with rotary shaking at 200 rpm. The cells were harvested by centrifugation at 5000 rpm, and washed twice with potassium phosphate buffer (0.1 M, pH 7.0).

Achmatowicz rearrangement in vivo

The reaction was performed at 30 °C in 1 ml of potassium phosphate buffer (0.1 M, pH 7.0) containing fresh recombinant *E. coli* cells (0.1 g), the substrate **1a** (1 mg) with shaking at 200 rpm for 7 h. The reaction was terminated by extraction with ethyl acetate (1:1, v/v). The samples were analyzed with chiral GC to determine the yield.

Redox isomerisation in vivo

The reaction was performed at 30 °C in 1 ml of potassium phosphate buffer (0.1 M, pH 7.0) containing fresh recombinant *E. coli* cells (0.1 g), the substrate pyranone (1 mg) with shaking at 200 rpm for 4 h. The reaction was terminated by extraction with ethyl acetate. The combined organic layer was dried over anhydrous Na_2SO_4 and concentrated under reduced pressure. The samples were analyzed with chiral GC to determine the yield and chiral HPLC to determine the enantiomeric excess.

Artificial pathway in vivo

The reaction was performed at 30 °C in 1 ml of potassium phosphate buffer (0.1 M, pH 7.0) containing fresh recombinant *E. coli* cells (0.1 g), the substrate (1 mg) and isopropanol (2ul) with shaking at 200 rpm for 20 h. The reaction was terminated by extraction with ethyl acetate. The combined organic layer was dried over anhydrous Na_2SO_4 and concentrated under reduced pressure. The samples were analyzed with chiral GC to determine the yield and chiral HPLC to determine the enantiomeric excess. The products were purified by silica gel column chromatography, eluted with cyclohexane/ethyl acetate (1: 1), and identified by NMR analysis. Reactions of 20 ml scale or multiple reactions were performed to accumulate enough products for the analysis when it is necessary.

Procedures & analytical data

Lactones

(*S*)-5-Hydroxy-6,6-dimethyl-5,6-dihydro-2*H*-pyran-2-one (3a)

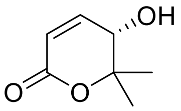

3a

According to the general procedure, **1a** (10 mg, 79.3 µmol) was reacted in presence of SMO, ReADH, and LkADH in *E. coli* BL21(DE3) Δ*nemA* (0.1 g, fresh weight). The residue was extracted and purified by column chromatography (SiO_2, hexane: ethyl acetate, 1:1) yielding (*S*)-**3a** (10.3 mg, 72.4 µmol, 91%, 99% *ee*) as colorless oil. **R_f** 0.34 (hexane/ethyl acetate 1/1). **^{1}H-NMR** (400 MHz, $CDCl_3$): δ [ppm] = 6.81 (dd, *J* = 9.8 Hz, *J* = 4.0 Hz, 1H), 6.05 (dd, *J* = 9.8 Hz, *J* = 1.3 Hz, 1H), 4.20 (s, 1H), 2.15 (br, 1H), 1.46 (s, 6H). **^{13}C-NMR** (100 MHz, $CDCl_3$): δ [ppm] = 162.9, 145.4, 121.6, 83.4, 68.6, 26.4, 21.8. **HPLC** (*Chiralpak AS*, hexane/isopropanol 9/1, 0.8 mL/min, 210 nm): t_R ((*S*)-**2f**) = 34.2 min, t_R ((*R*)-**2f**) = 37.5 min (e.r. > 99:1).

(*S*)-9-Hydroxy-5-oxaspiro[3.5]non-7-en-6-one (3b)

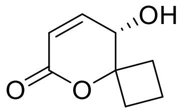

3b

R_f 0.36 (hexane/ethyl acetate 1/1). **^{1}H-NMR** (400 MHz, $CDCl_3$): δ [ppm] = 6.91 (dd, *J* = 9.6 Hz, *J* = 4.8 Hz, 1H), 6.05 (dd, *J* = 9.7 Hz, *J* = 0.7 Hz, 1H), 4.25 (q, 1H), 2.53-2.61 (m, 1H), 2.25-2.38 (m, 2H), 2.11-2.19 (m, 1H), 1.94-2.04 (m, 2H), 1.64-1.76 (m, 1H). **^{13}C-NMR** (100 MHz, $CDCl_3$): δ [ppm] = 144.1, 122.6, 65.5, 32.0, 29.2, 12.5. **HPLC** (*Chiralpak AS*, hexane/isopropanol 9/1, 0.8 mL/min, 210 nm): t_R ((*S*)-**2c**) = 39.3 min, t_R ((*R*)-**2c**) = 60.1 min (e.r. = 70:30).

(*S*)-10-Hydroxy-6-oxaspiro[4.5]dec-8-en-7-one (3c)

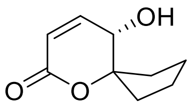

3c

R_f 0.36 (hexane/ethyl acetate 1/1). **^{1}H-NMR** (400 MHz, $CDCl_3$): δ [ppm] = 6.93 (dd, *J* = 9.7 Hz, *J* = 4.6 Hz, 1H), 6.04 (dd, *J* = 9.7 Hz, *J* = 0.9 Hz, 1H), 4.16-4.19 (q, 1H), 2.18 (d, *J* = 8.4 Hz, 1H), 1.66-2.05 (m, 8H). **^{13}C-NMR** (100 MHz, $CDCl_3$): δ [ppm] = 163.5, 145.8, 122.2, 94.3, 67.0, 37.7, 33.9, 24.4, 24.0. **HPLC** (*Chiralpak AS*, hexane/isopropanol 9/1, 0.8 mL/min, 210 nm): t_R ((*S*)-**2b**) = 33.7 min, t_R ((*R*)-**2f**) = 45.7 min (e.r. = 80:20).

Starting materials

2-(furan-2-yl)propanol (1a)

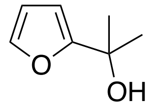

1a

To a solution of acetylfuran (1.10 g, 10.0 mmol) in anhydrous THF (20 mL) methylmagnesium bromide (2.0 equiv, 20.0 mmol) was added at 0°C under argon, and the mixture was allowed to warm to room temperature overnight. Saturated aqueous NH_4Cl solution (10 mL) was added, the mixture was extracted with diethyl ether (3 x 15 mL), the combined organic layers dried over Na_2SO_4, and concentrated under reduced pressure. The residue was purified by column chromatography (SiO_2, n-pentane:diethyl ether, 10:1). **R_f** (pentane:diethyl ether, 5:1): 0.40. **^{1}H-NMR** (400 MHz, $CDCl_3$): δ [ppm] = 7.34 (dd, ^3J = 1.8 Hz, ^3J = 0.8 Hz, 1H), 6.29 (dd, ^3J = 3.2 Hz, ^3J = 1.8 Hz, 1H), 6.18 (dd, ^3J = 3.2 Hz, ^3J = 0.8 Hz, 1H), 1.94 (br, 1H), 1.58 (s, 6H). **^{13}C-NMR** (400 MHz, $CDCl_3$): δ [ppm] = 160.2, 141.5, 110.0, 103.6, 68.8, 28.7. **GC** t_R = 17.4 min.

1-(furan-2-yl)cyclobutanol (1b)

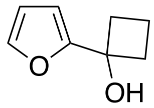

1b

To a solution of furan (680 mg, 10.0 mmol) in anhydrous THF (20 mL) was added n-BuLi (2.5M, 1.0 equiv, 10.0 mmol) and N,N,N´,N´-tetramethylethylenediamine (1.0 equiv, 10.0mmol) at –78 °C under argon, and the mixture was stirred for 1 h. Cyclobutanone (0.2 equiv, 2 mmol) was added at –78 °C, and the reaction mixture was stirred for 1 h at –78 °C. The reaction was quenched with ice water (10 mL), extracted with diethyl ether (3 x 15 mL), dried over Na_2SO_4, and concentrated under reduced pressure. The residue was purified by column chromatography (SiO2, n-pentane: diethyl ether = 10:1) giving as colorless oil. **R_f** (pentane:diethyl ether, 5:1): 0.40. **^{1}H-NMR** (400 MHz, $CDCl_3$): δ [ppm] = 7.37 (dd, J = 1.8 Hz, J = 0.8 Hz, 1H), 6.32 (dd, J = 3.2 Hz, J = 1.8 Hz, 1H), 6.27 (dd, J = 3.2 Hz, J = 0.8 Hz, 1H), 2.46-2.53 (m, 2H), 2.42 (br, 1H), 2.27-2.35 (m, 2H), 1.81-1.91 (m, 1H), 1.61-1.71 (m, 1H). **^{13}C-NMR** (400 MHz, $CDCl_3$): δ [ppm] = 158.2, 142,1, 110.0, 104.9, 72.2 35.7, 12.7. **GC** t_R = 28.3 min.

1-(furan-2-yl)cyclopentanol (1c)

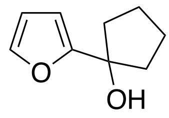

1c

In first flask, cyclopentanone (0.2 equiv, 2mmol) was added to a stirred suspension of magnesium sulfate (1.0 equiv, 10 mmol) in anhydrous THF (20ml) at room temperature for

1h. In the second flask, to a solution of furan (680 mg, 10.0 mmol) in anhydrous THF (20 mL) was added n-BuLi (2.5M, 1.0 equiv, 10.0 mmol) and N,N,N´,N´-tetramethyl-ethylenediamine (1.0 equiv, 10.0mmol) at –78 °C under argon for 1 h. the mixture was then transferred dropwise into the first flask at -78 °C. The resulting mixture was allowed to warm to room temperature overnight. The reaction was quenched with ice water (10 mL), extracted with diethyl ether (3 x 15 mL), dried over Na_2SO_4, and concentrated under reduced pressure. The residue was purified by column chromatography (SiO2, n-pentane: diethyl ether = 10:1) giving as colorless oil. **R*f*** (pentane:diethyl ether, 5:1): 0.40. **^{1}H-NMR** (400 MHz, $CDCl_3$): δ [ppm] = 7.33 (dd, *J* = 1.8, *J* = 0.8, 1H), 6.29 (dd, *J* = 3.2 Hz, *J* = 1.8 Hz, 1H), 6.19 (d, *J* = 3.2 Hz, *J* = 1.8 Hz, 1H), 1.89-2.03 (m, 6H), 1.84-1.93 (m, 1H), 1.70-1.74 (m, 2H). **^{13}C-NMR** (100 MHz, $CDCl_3$): δ [ppm] = 159.3, 141,5, 110.0, 104.1, 79.5, 39.6, 23.6. **^{13}C-NMR** (400 MHz, $CDCl_3$): δ [ppm] = 159.3, 141.5, 110.0, 104.1, 79.5, 39.6, 23.6. **GC** t_R = 31.8 min.

1-(furan-2-yl)cyclohexanol (1d)

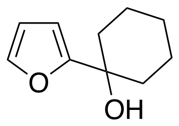

1d

1d was synthesized in the same fashion as **1c**. **R*f*** (pentane:diethyl ether, 5:1): 0.40. **^{1}H-NMR** (400 MHz, $CDCl_3$): δ [ppm] = 7.34 (dd, *J* = 1.8 Hz, *J* = 0.8 Hz, 1H), 6.31 (dd, *J* = 3.2 Hz, *J* = 1.8 Hz, 1H), 6.20 (dd, *J* = 3.2 Hz, *J* = 0.8 Hz, 1H), 1.94-2.00 (m, 2H), 1.90 (s, 1H), 1.80-1.86 (m, 2H), 1.67-1.76 (m, 6H). **^{13}C-NMR** (400 MHz, $CDCl_3$): δ [ppm] = 160.0, 141.4, 110.0, 104.4, 70.1, 36.6, 25.5, 22.2. **GC** t_R = 34.1 min.

NMR spectra of the compounds

(*S*)-5-Hydroxy-6,6-dimethyl-5,6-dihydro-2*H*-pyran-2-one (3a)

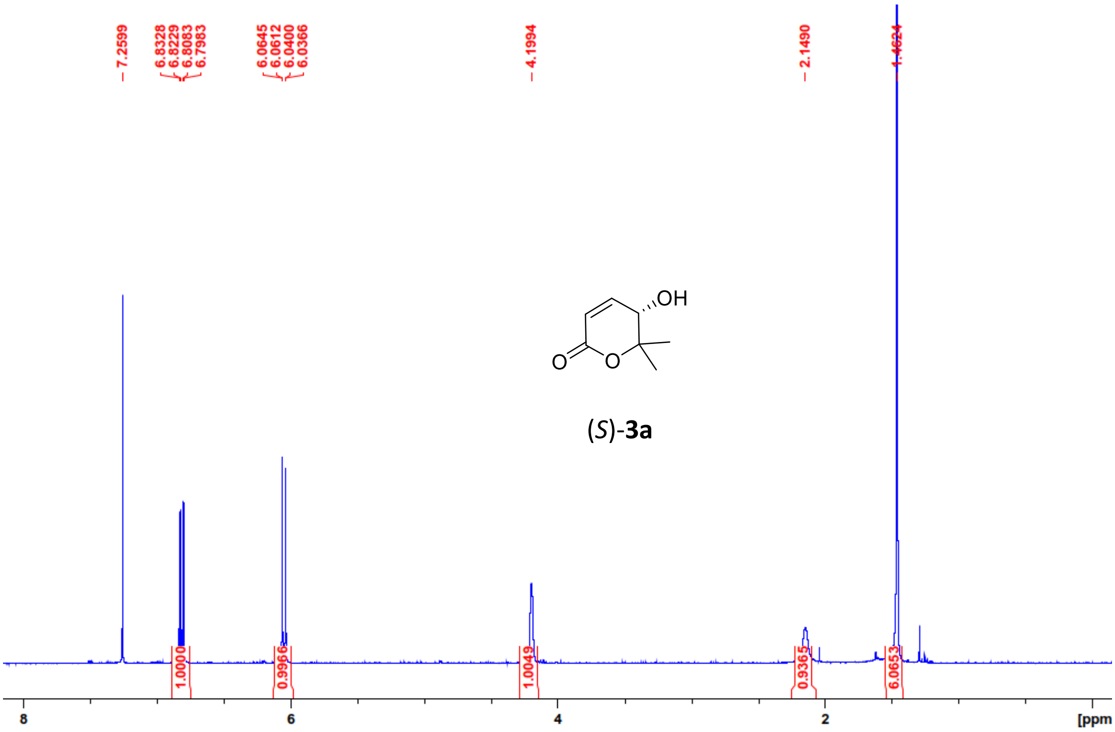

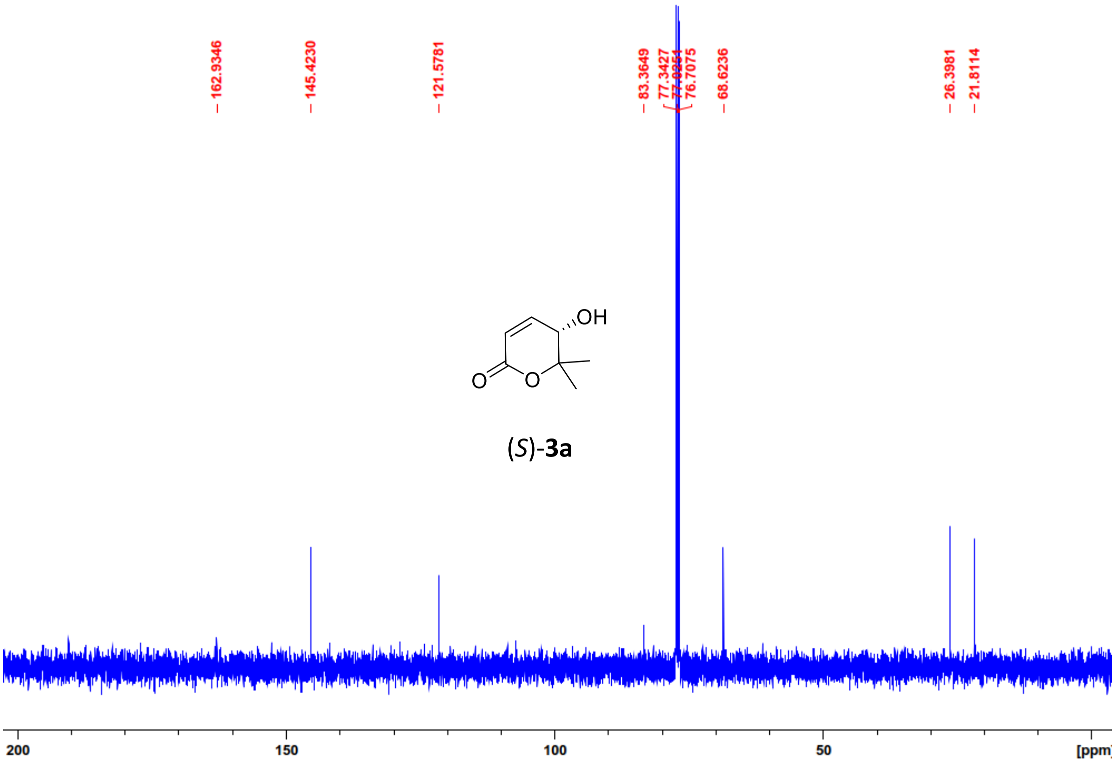

(*S*)-9-Hydroxy-5-oxaspiro[3.5]non-7-en-6-one (3b)

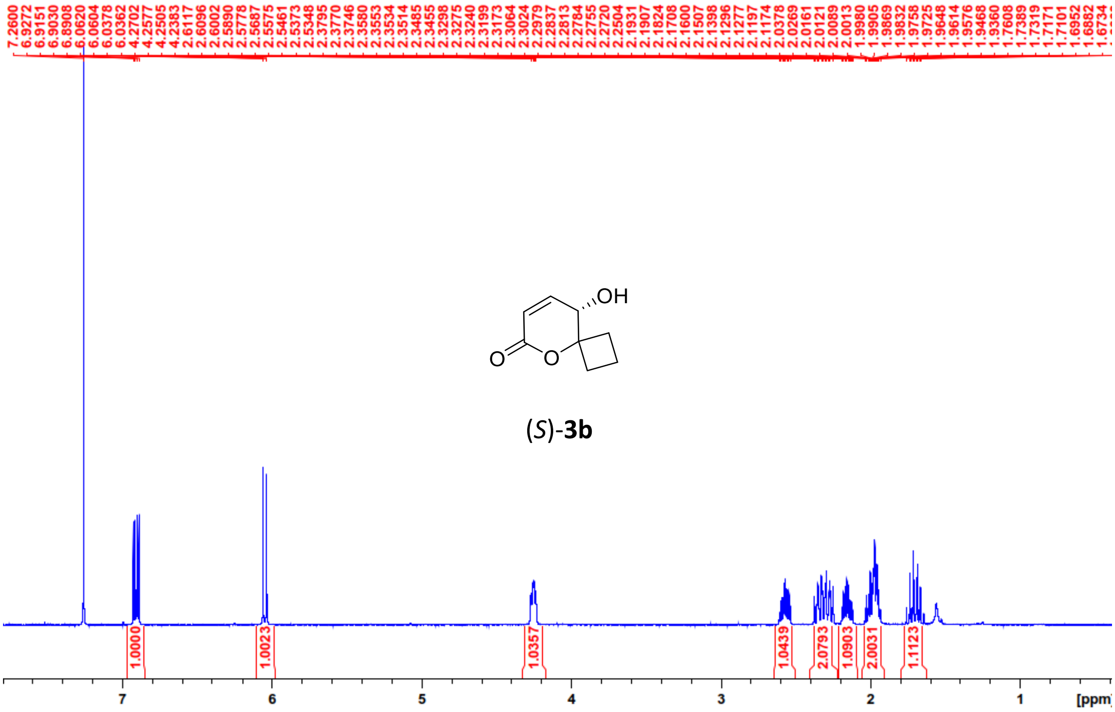

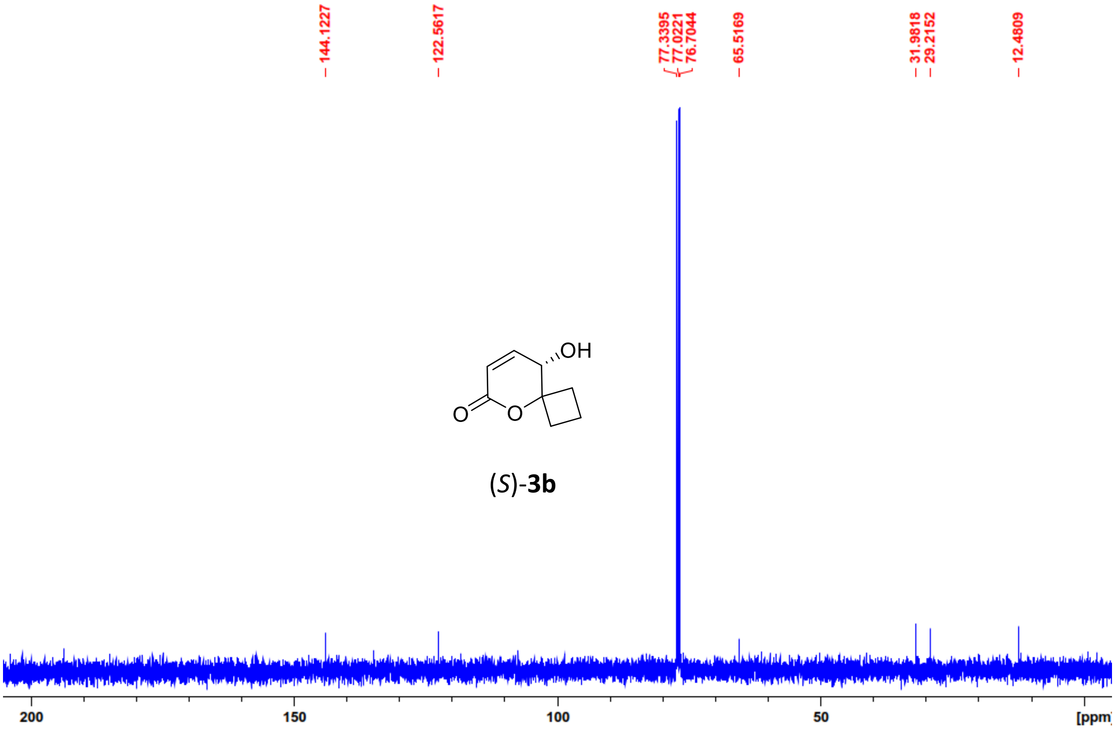

(*S*)-10-Hydroxy-6-oxaspiro[4.5]dec-8-en-7-one (3c)

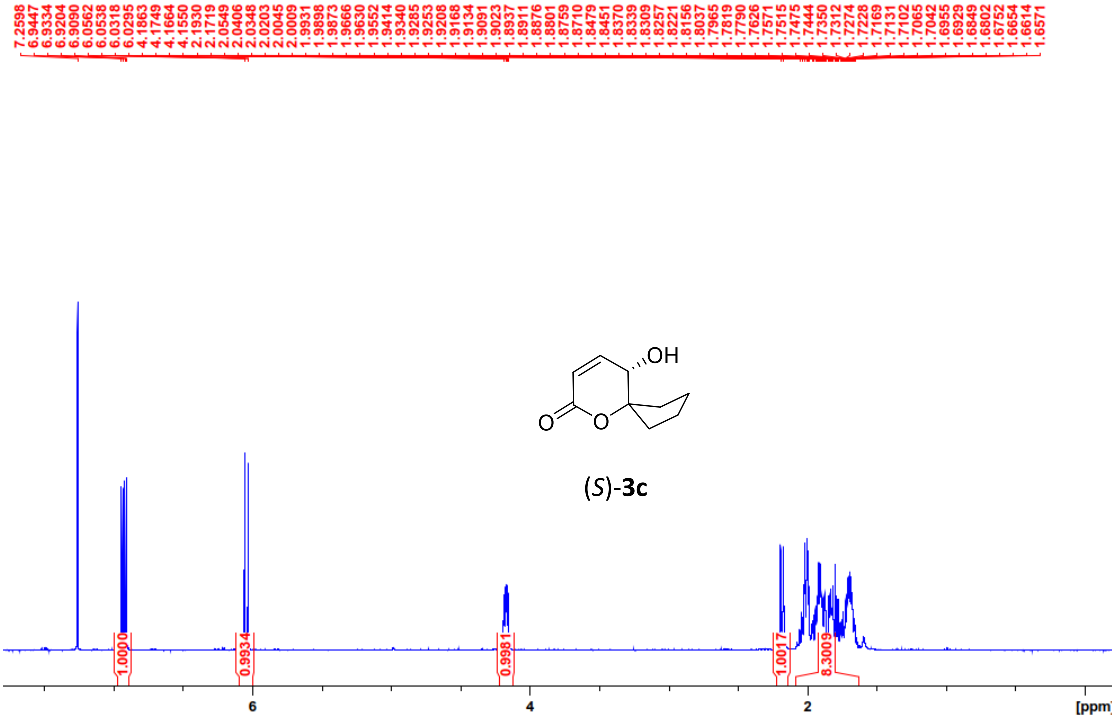

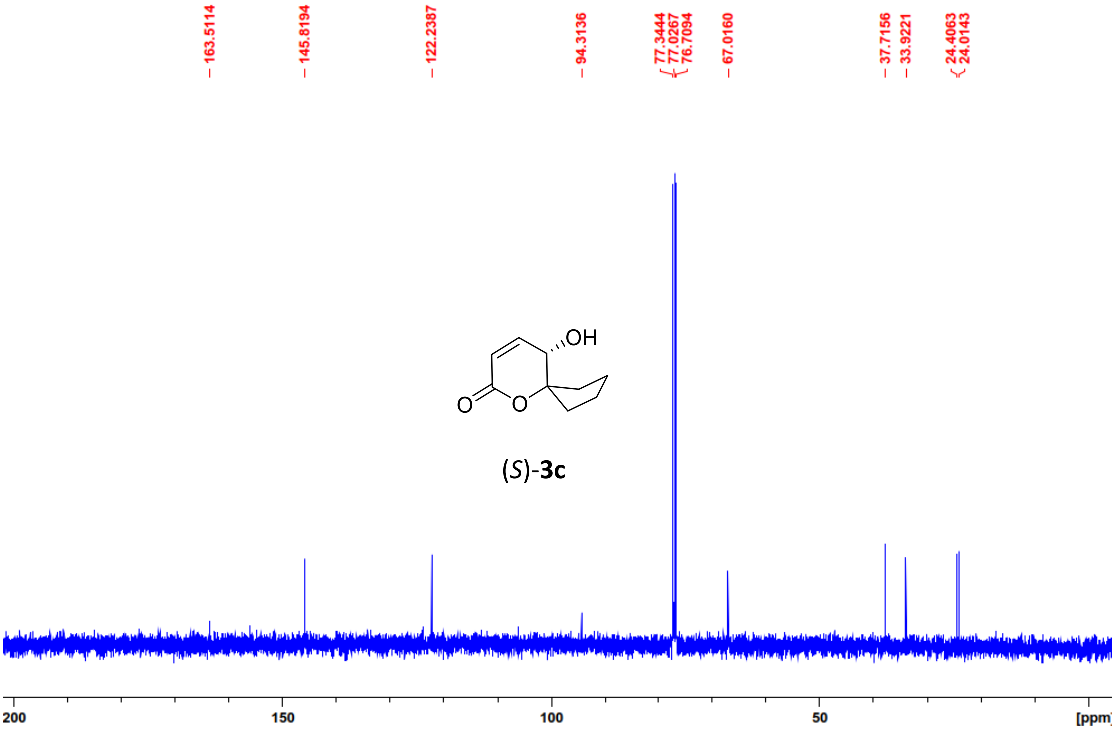

GC traces

(*S*)-5-Hydroxy-6,6-dimethyl-5,6-dihydro-2*H*-pyran-2-one (3a)

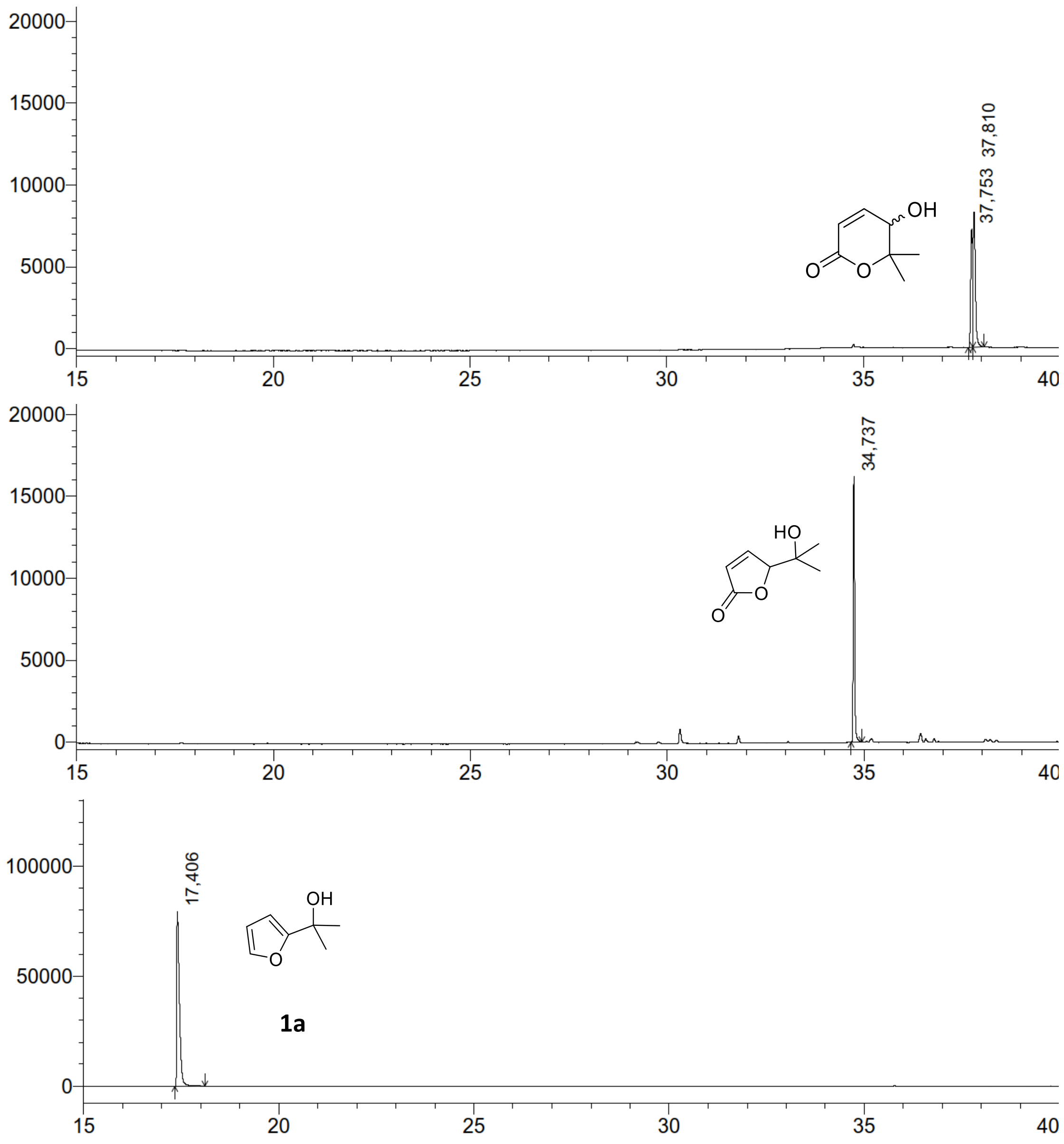

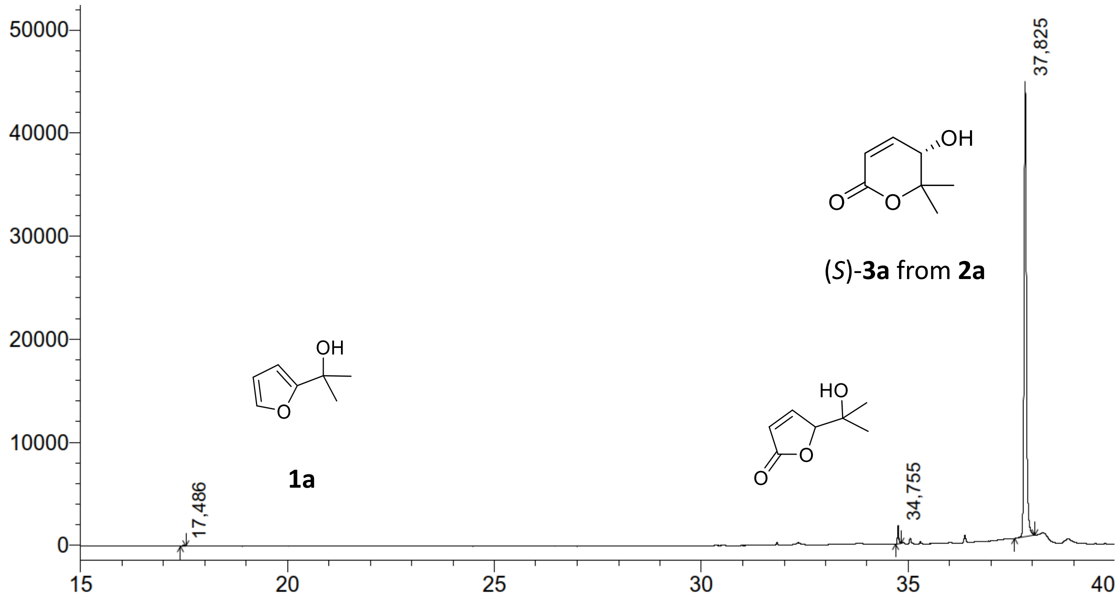

FID1

Peak#	Ret. Time	Area	Height	Conc.
1	17,486	371	133	0,208
2	34,755	4223	1755	2,373
3	37,825	173411	44108	97,419
Total		178006	45996	

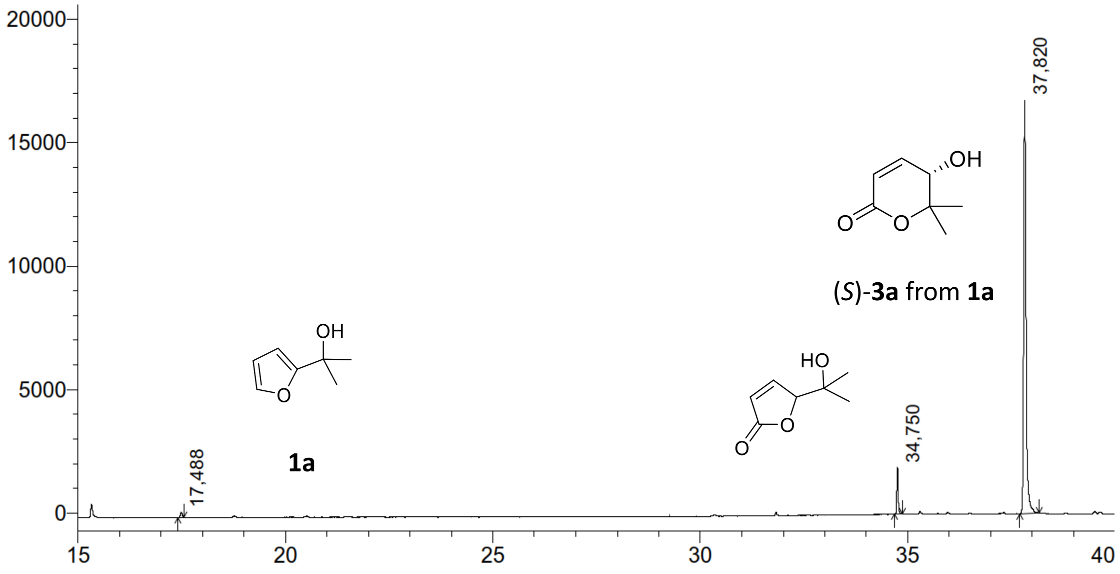

FID1

Peak#	Ret. Time	Area	Height	Conc.
1	17,488	619	200	0,862
2	34,750	4682	1868	6,524
3	37,820	66456	16668	92,613
Total		71756	18735	

(*S*)-9-Hydroxy-5-oxaspiro[3.5]non-7-en-6-one (3b)

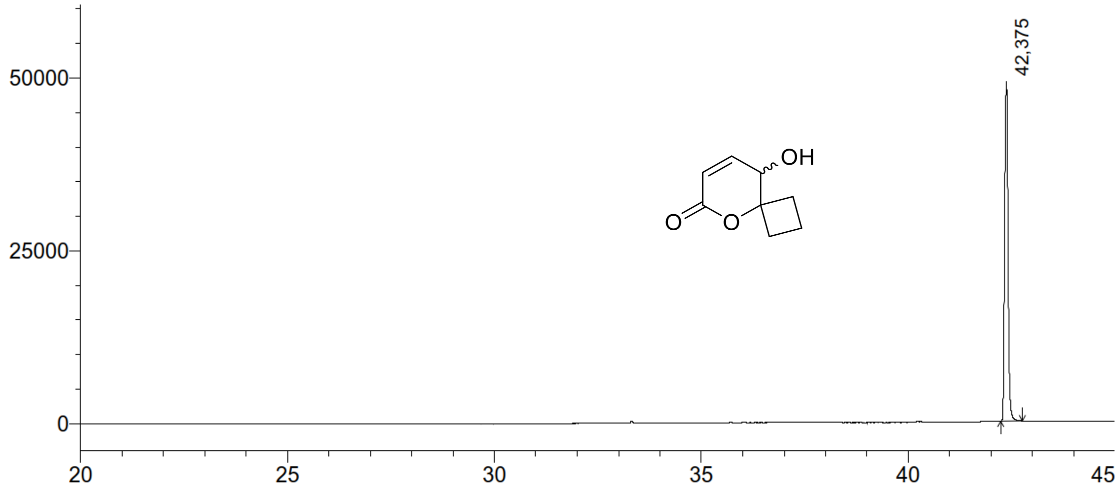

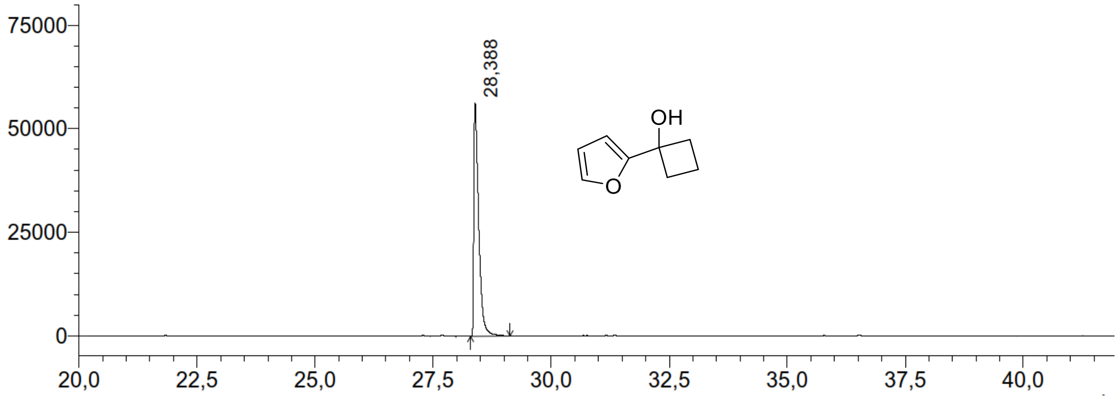

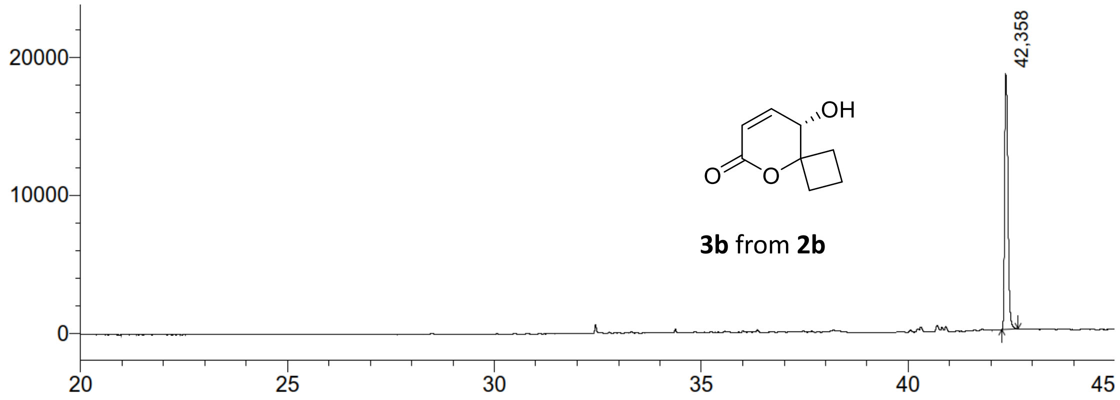

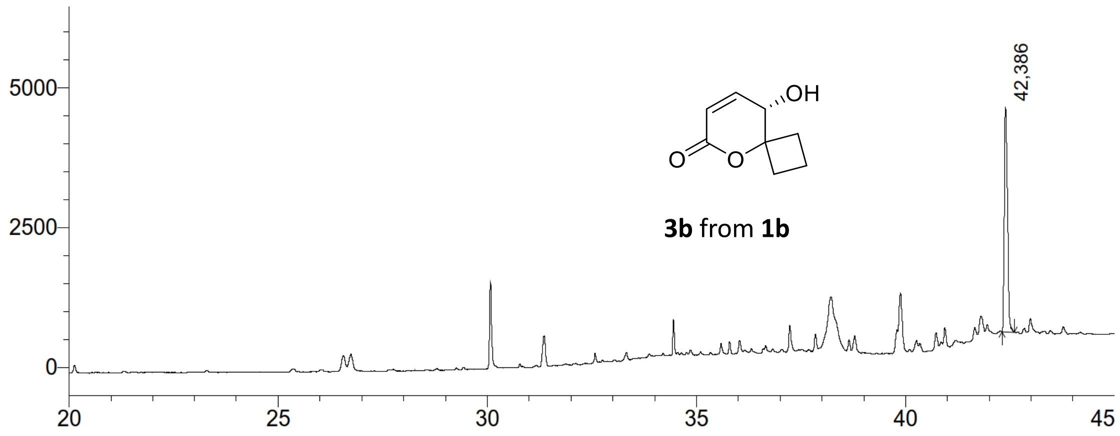

(*S*)-10-Hydroxy-6-oxaspiro[4.5]dec-8-en-7-one (3c)

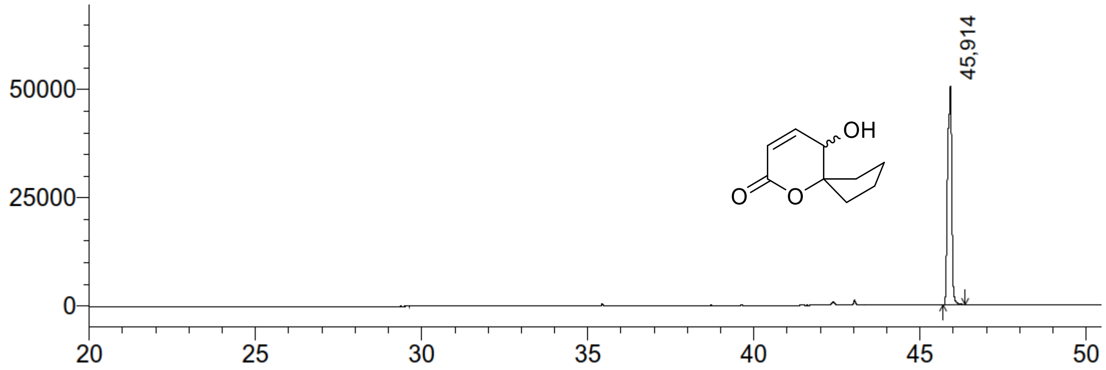

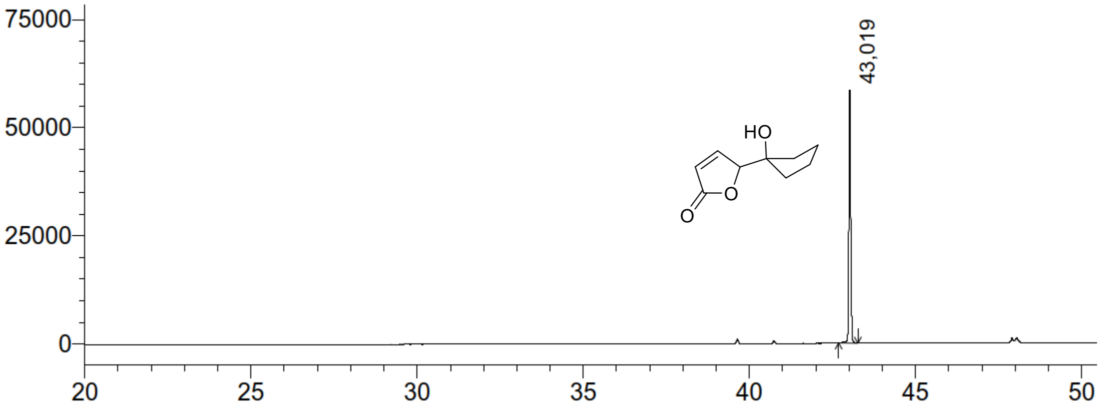

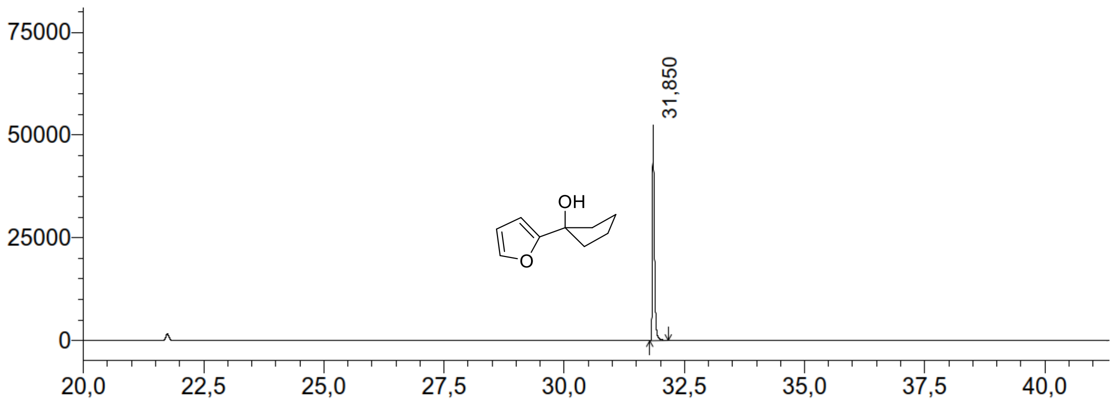

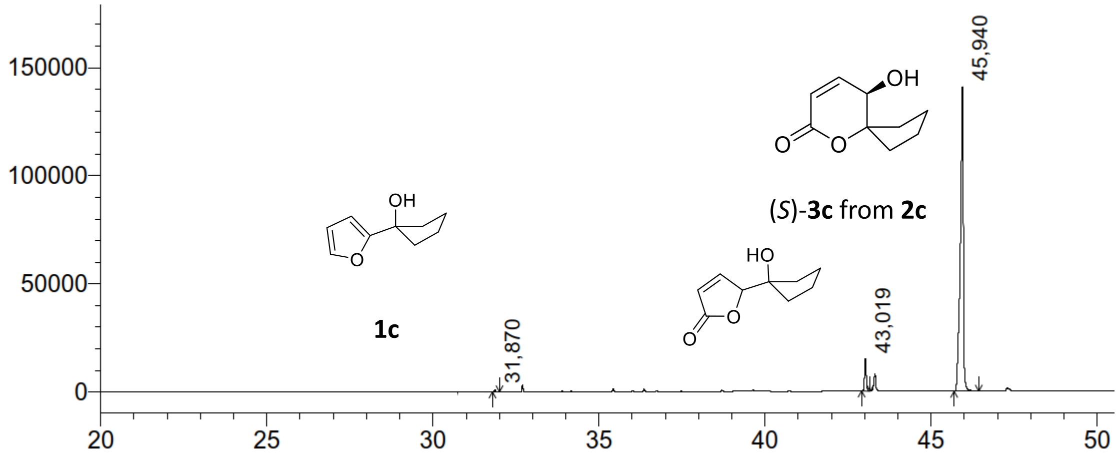

FID1

Peak#	Ret. Time	Area	Height	Conc.
1	31,870	3029	1087	0,323
2	43,019	51776	14866	5,528
3	45,940	881734	140617	94,148
Total		936538	156570	

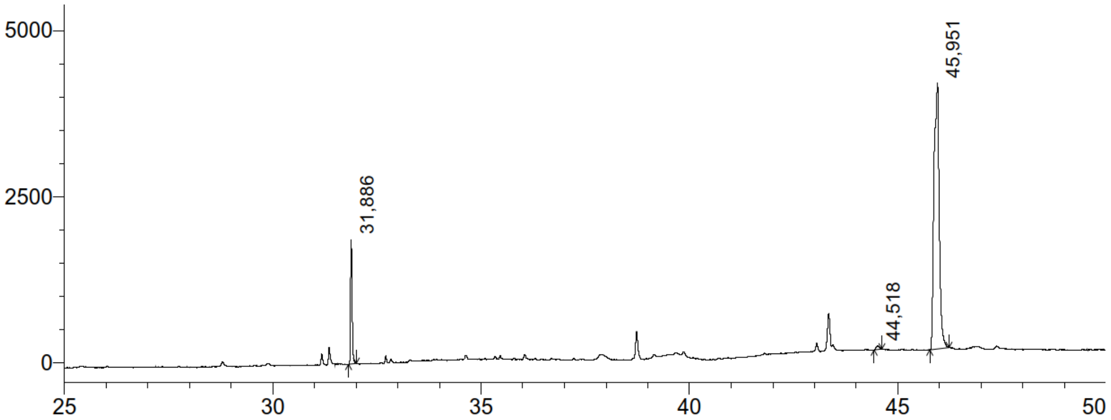

FID1

Peak#	Ret. Time	Area	Height	Conc.
1	31,886	4967	1867	12,367
2	44,518	293	53	0,729
3	45,951	34903	4003	86,904
Total		40162	5923	

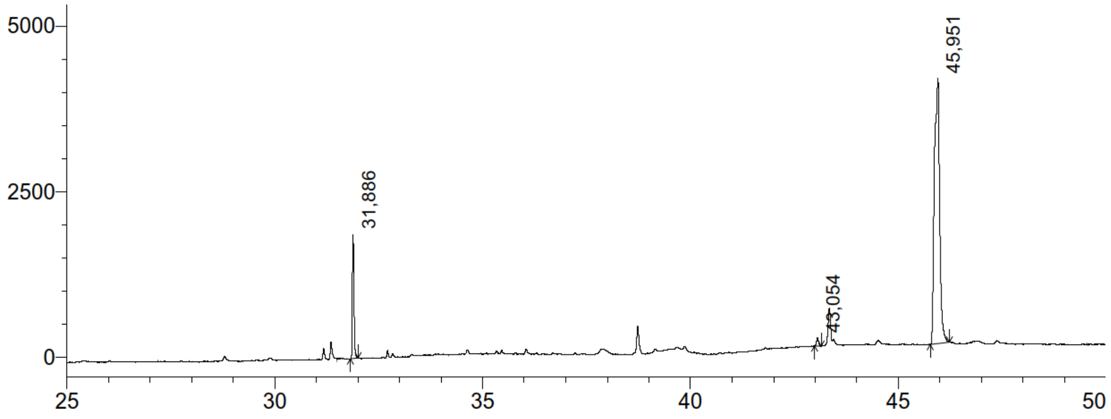

FID1

Peak#	Ret. Time	Area	Height	Conc.
1	31,886	4967	1867	12,321
2	43,054	442	124	1,097
3	45,951	34903	4003	86,582
Total		40312	5993	

(*S*)-5-hydroxy-1-oxaspiro[5.5]undec-3-en-2-one (3d)

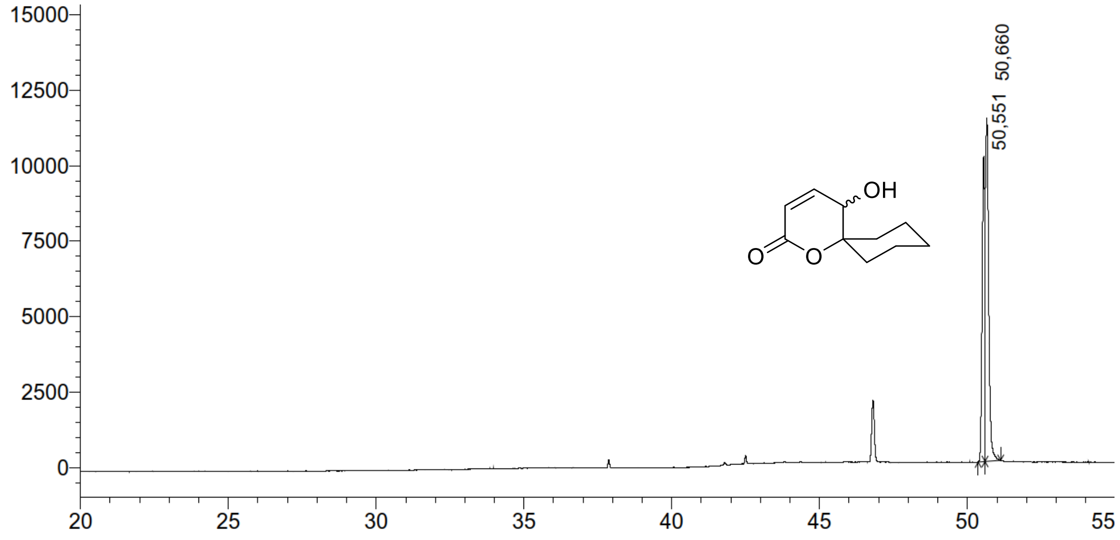

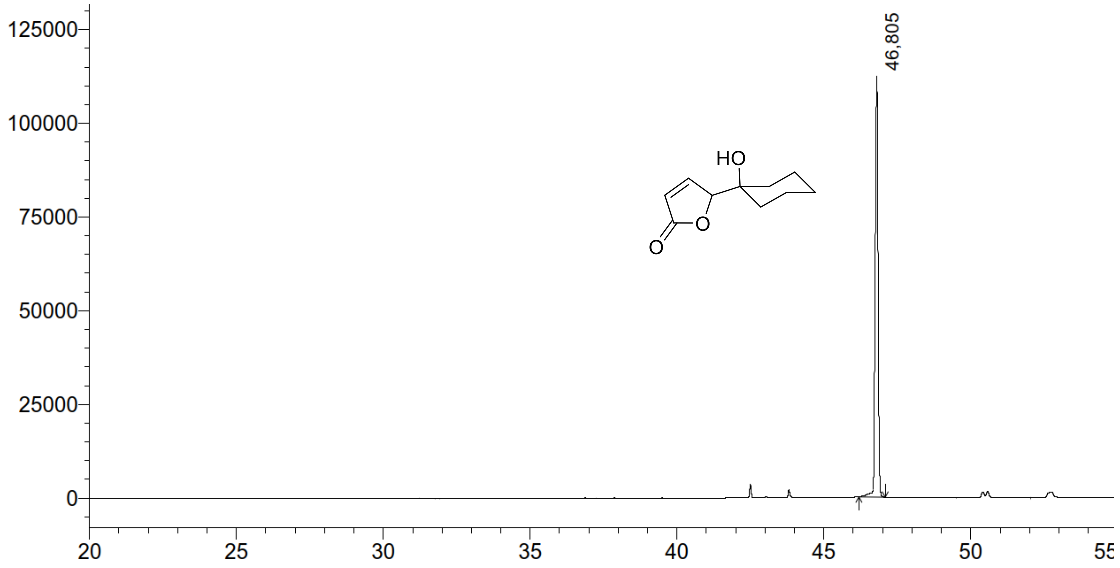

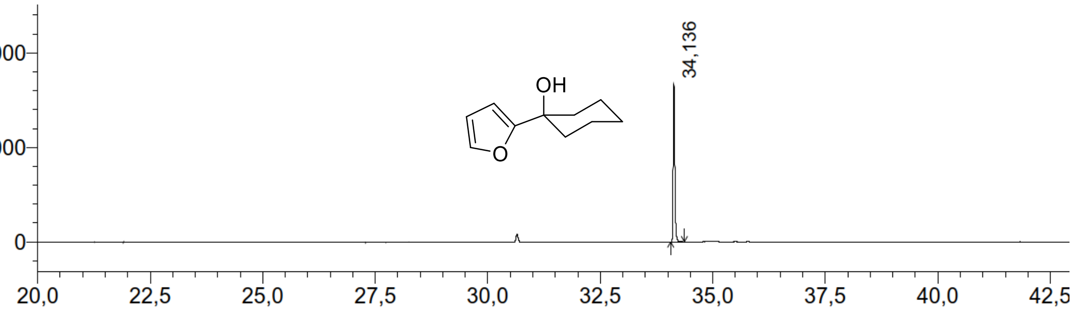

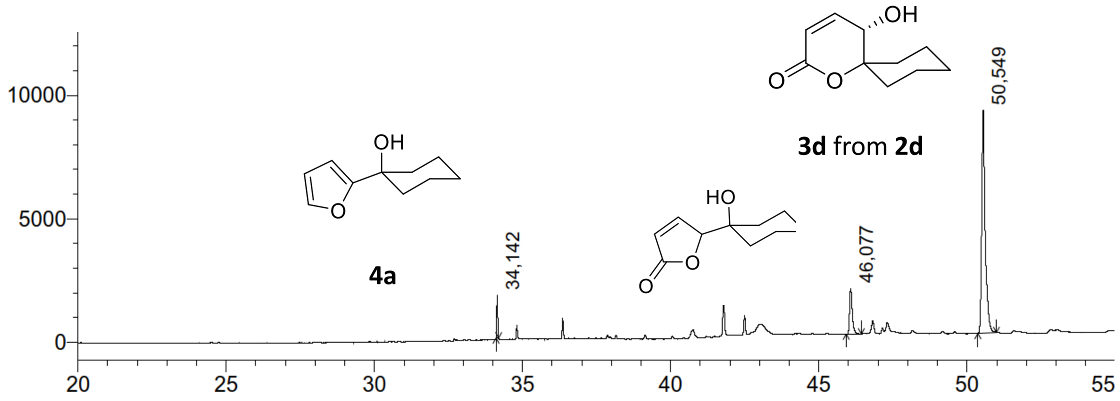

FID1

Peak#	Ret. Time	Area	Height	Conc.
1	34,142	3695	1705	4,091
2	46,077	11106	1850	12,296
3	50,549	75525	9027	83,614
Total		90326	12582	

HPLC traces

(*S*)-5-Hydroxy-6,6-dimethyl-5,6-dihydro-2*H*-pyran-2-one (1d)

```
Acq. Operator    : SYSTEM
Sample Operator : SYSTEM
Acq. Instrument : HPLC-1 NP                        Location :   61
Injection Date  : 9.3.2019 9:03:53
                                                Inj Volume : 2.000 µl
Acq. Method      : C:\Chem32\2\Methods\DEF_LC1.M
Last changed     : 9.3.2019 8:49:11 by SYSTEM
                   (modified after loading)
Analysis Method : D:\LCData\Jäger\Method\Jäger.M
Last changed     : 11.3.2019 9:26:03 by SYSTEM
                   (modified after loading)
Additional Info : Peak(s) manually integrated
```

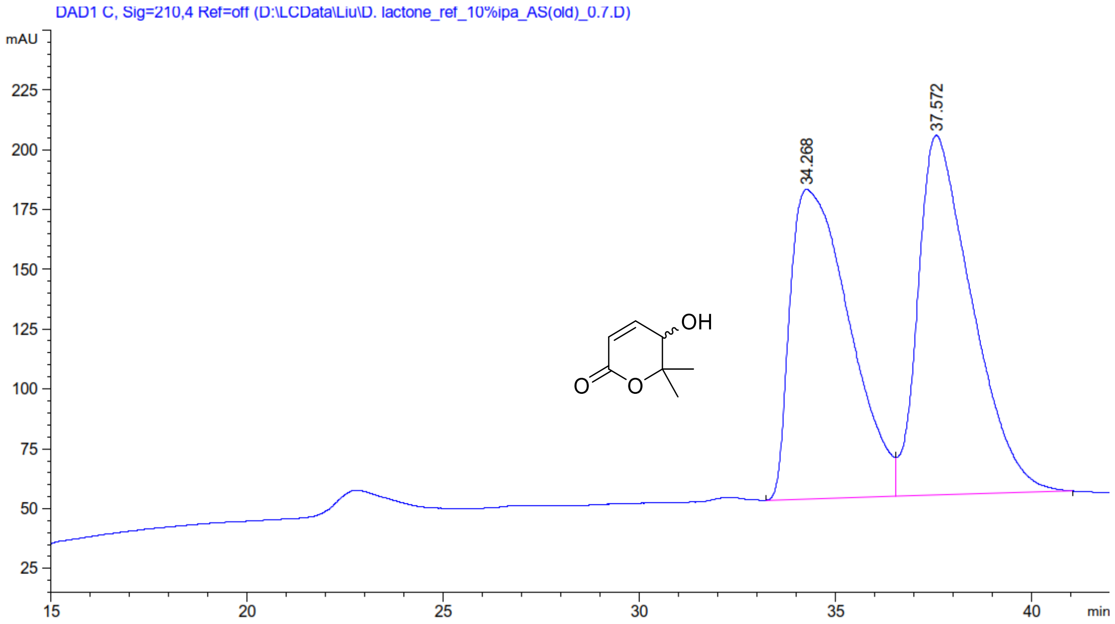

Acq. Operator : SYSTEM
Sample Operator : SYSTEM
Acq. Instrument : HPLC-1 NP Location : 62
Injection Date : 9.3.2019 9:51:28
Inj Volume : 2.000 µl
Acq. Method : C:\Chem32\2\Methods\DEF_LC1.M
Last changed : 9.3.2019 8:49:11 by SYSTEM
(modified after loading)
Analysis Method : D:\LCData\Jäger\Method\Jäger.M
Last changed : 11.3.2019 9:25:34 by SYSTEM
(modified after loading)
Additional Info : Peak(s) manually integrated

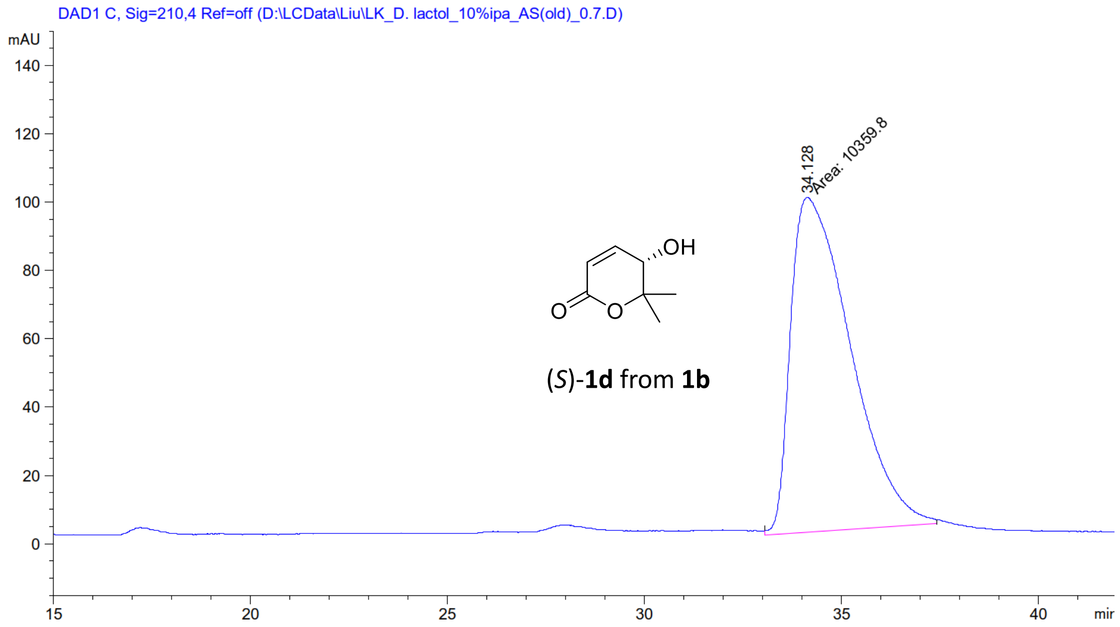

Acq. Operator : SYSTEM
Sample Operator : SYSTEM
Acq. Instrument : HPLC-1 NP Location : 63
Injection Date : 10.3.2019 8:11:55
Inj Volume : 2.000 µl
Acq. Method : C:\Chem32\2\Methods\DEF_LC1.M
Last changed : 9.3.2019 8:49:11 by SYSTEM
(modified after loading)
Analysis Method : D:\LCData\Jäger\Method\Jäger.M
Last changed : 11.3.2019 9:26:41 by SYSTEM
(modified after loading)
Additional Info : Peak(s) manually integrated

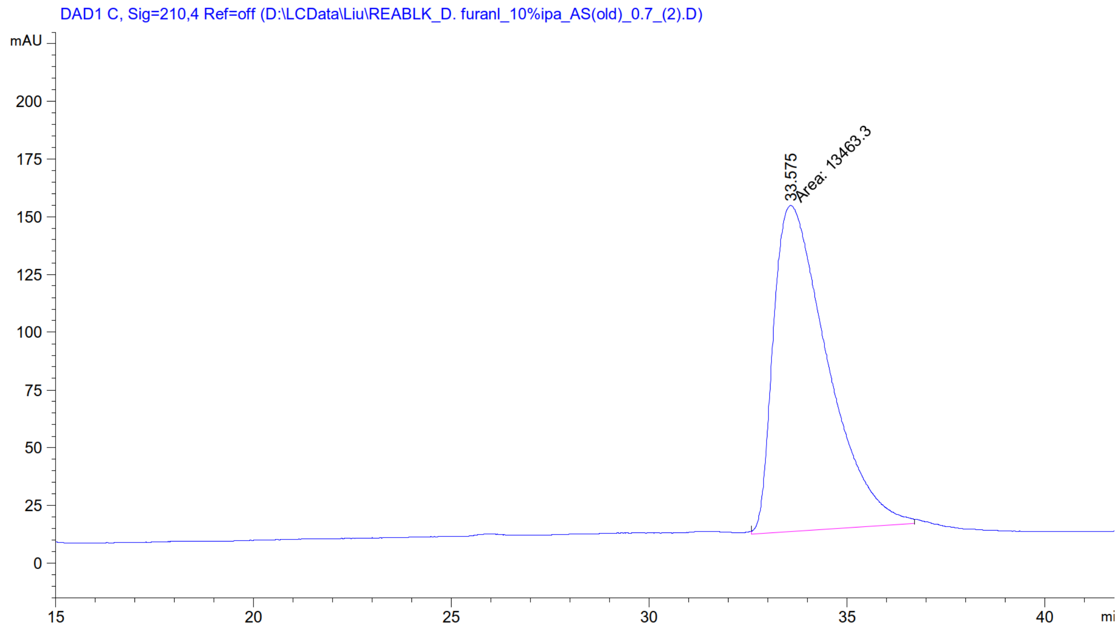

(*S*)-9-Hydroxy-5-oxaspiro[3.5]non-7-en-6-one (2d)

```
Acq. Operator   : SYSTEM
Sample Operator : SYSTEM
Acq. Instrument : HPLC-1 NP                          Location :   71
Injection Date  : 19.3.2019 17:28:20
                                                 Inj Volume : 2.000 µl
Different Inj Volume from Sample Entry!  Actual Inj Volume : 5.000 µl
Acq. Method     : C:\Chem32\2\Methods\DEF_LC1.M
Last changed    : 19.3.2019 15:55:37 by SYSTEM
                  (modified after loading)
Analysis Method : D:\LCData\Jäger\Method\Jäger.M
Last changed    : 19.3.2019 20:20:07 by SYSTEM
                  (modified after loading)
Additional Info : Peak(s) manually integrated
```

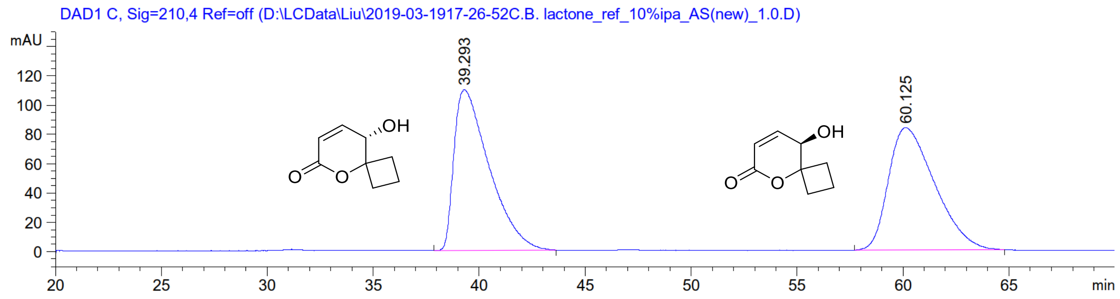

```
Acq. Operator    : SYSTEM
Sample Operator  : SYSTEM
Acq. Instrument  : HPLC-1 NP                    Location :   72
Injection Date   : 19.3.2019 16:14:23
                                              Inj Volume : 2.000 µl
Different Inj Volume from Sample Entry!  Actual Inj Volume : 5.000 µl
Acq. Method      : C:\Chem32\2\Methods\DEF_LC1.M
Last changed     : 19.3.2019 15:55:37 by SYSTEM
                   (modified after loading)
Analysis Method  : D:\LCData\Jäger\Method\Jäger.M
Last changed     : 19.3.2019 20:20:07 by SYSTEM
                   (modified after loading)
Additional Info  : Peak(s) manually integrated
```

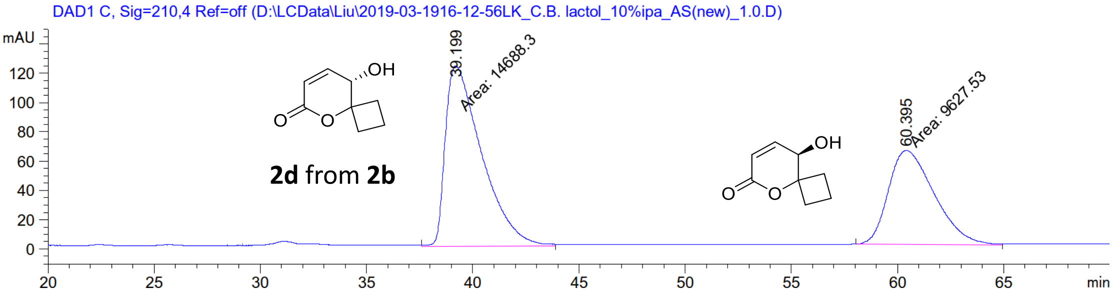


```
Acq. Operator    : SYSTEM
Sample Operator  : SYSTEM
Acq. Instrument  : HPLC-1 NP                    Location :   73
Injection Date   : 19.3.2019 19:22:32
                                              Inj Volume : 2.000 µl
Different Inj Volume from Sample Entry!  Actual Inj Volume : 5.000 µl
Acq. Method      : C:\Chem32\2\Methods\DEF_LC1.M
Last changed     : 19.3.2019 15:55:37 by SYSTEM
                   (modified after loading)
Analysis Method  : D:\LCData\Jäger\Method\Jäger.M
Last changed     : 19.3.2019 20:20:07 by SYSTEM
                   (modified after loading)
Additional Info  : Peak(s) manually integrated
```

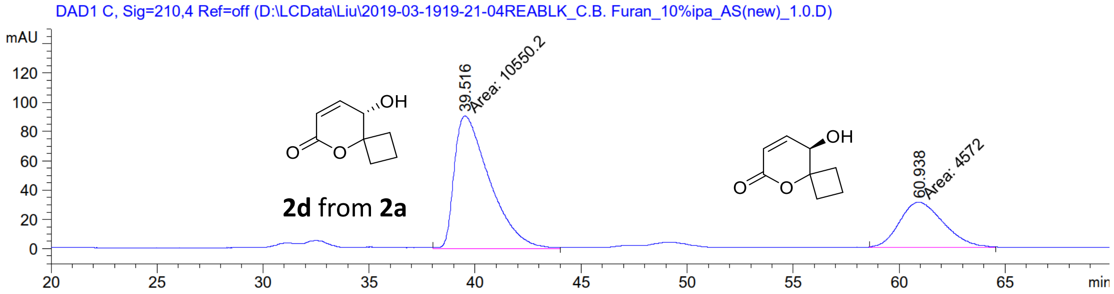

(*S*)-10-Hydroxy-6-oxaspiro[4.5]dec-8-en-7-one (3d)

```
Acq. Operator   : SYSTEM
Sample Operator : SYSTEM
Acq. Instrument : HPLC-1 NP                        Location :   81
Injection Date  : 21.3.2019 13:40:30
                                                 Inj Volume : 2.000 µl
Different Inj Volume from Sample Entry!  Actual Inj Volume : 4.000 µl
Acq. Method     : C:\Chem32\2\Methods\DEF_LC.M
Last changed    : 20.3.2019 13:28:12 by SYSTEM
                  (modified after loading)
Analysis Method : D:\LCData\Jäger\Method\Jäger.M
Last changed    : 21.3.2019 14:35:46 by SYSTEM
                  (modified after loading)
Additional Info : Peak(s) manually integrated
```

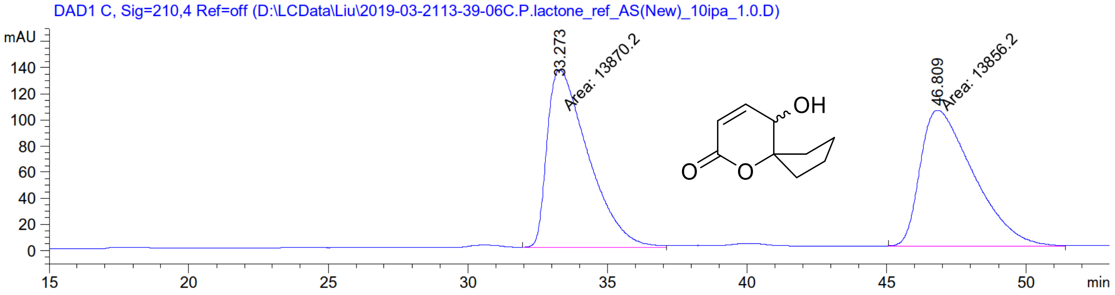


```
Acq. Operator   : SYSTEM
Sample Operator : SYSTEM
Acq. Instrument : HPLC-1 NP                        Location :   82
Injection Date  : 20.3.2019 13:29:48
                                                 Inj Volume : 2.000 µl
Acq. Method     : C:\Chem32\2\Methods\DEF_LC.M
Last changed    : 20.3.2019 13:28:12 by SYSTEM
                  (modified after loading)
Analysis Method : D:\LCData\Jäger\Method\Jäger.M
Last changed    : 21.3.2019 14:32:54 by SYSTEM
                  (modified after loading)
Additional Info : Peak(s) manually integrated
```

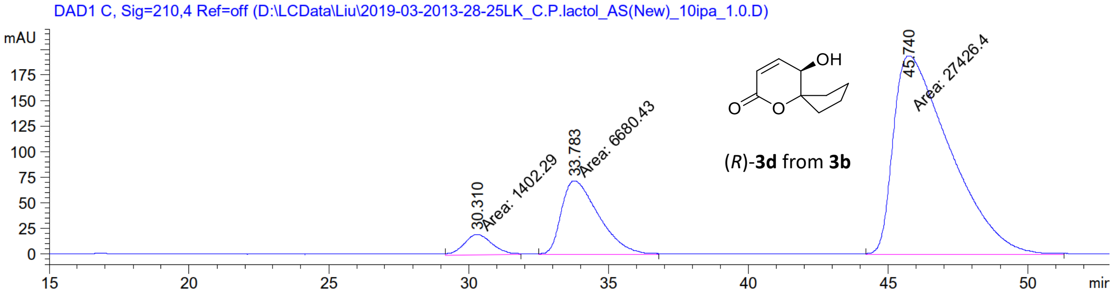

Acq. Operator : SYSTEM
Sample Operator : SYSTEM
Acq. Instrument : HPLC-1 NP Location : 83
Injection Date : 20.3.2019 15:26:02
Inj Volume : 2.000 µl
Different Inj Volume from Sample Entry! Actual Inj Volume : 5.000 µl
Acq. Method : C:\Chem32\2\Methods\DEF_LC.M
Last changed : 20.3.2019 13:28:12 by SYSTEM
(modified after loading)
Analysis Method : D:\LCData\Jäger\Method\Jäger.M
Last changed : 21.3.2019 14:34:00 by SYSTEM
(modified after loading)
Additional Info : Peak(s) manually integrated

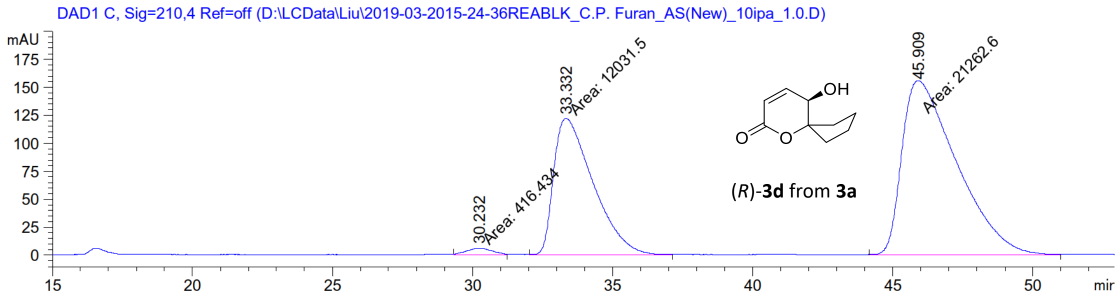

(S)-5-hydroxy-1-oxaspiro[5.5]undec-3-en-2-one (4d)

Acq. Operator : SYSTEM
Sample Operator : SYSTEM
Acq. Instrument : HPLC-1 NP Location : 91
Injection Date : 20.3.2019 9:32:34
Inj Volume : 2.000 µl
Different Inj Volume from Sample Entry! Actual Inj Volume : 5.000 µl
Acq. Method : C:\Chem32\2\Methods\DEF_LC1.M
Last changed : 20.3.2019 9:22:22 by SYSTEM
(modified after loading)
Analysis Method : D:\LCData\Jäger\Method\Jäger.M
Last changed : 20.3.2019 13:33:36 by SYSTEM
(modified after loading)
Additional Info : Peak(s) manually integrated

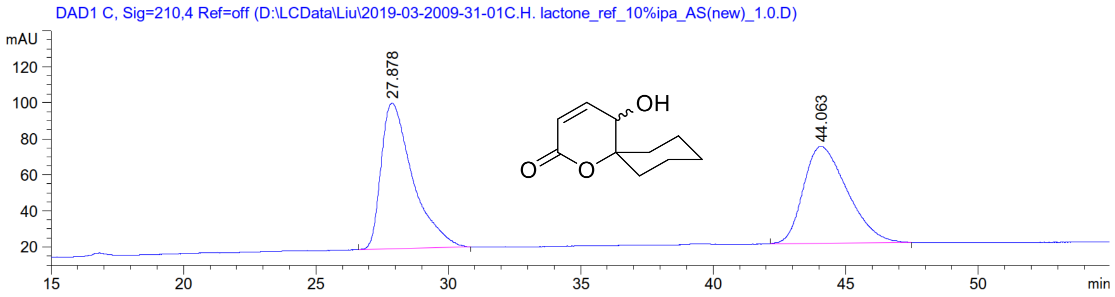

```
Acq. Operator    : SYSTEM
Sample Operator  : SYSTEM
Acq. Instrument  : HPLC-1 NP                        Location :   92
Injection Date   : 20.3.2019 10:30:23
                                                 Inj Volume : 2.000 µl
Different Inj Volume from Sample Entry!   Actual Inj Volume : 5.000 µl
Acq. Method      : C:\Chem32\2\Methods\DEF_LC1.M
Last changed     : 20.3.2019 9:22:22 by SYSTEM
                   (modified after loading)
Analysis Method  : D:\LCData\Jäger\Method\Jäger.M
Last changed     : 20.3.2019 13:34:46 by SYSTEM
                   (modified after loading)
Additional Info  : Peak(s) manually integrated
```

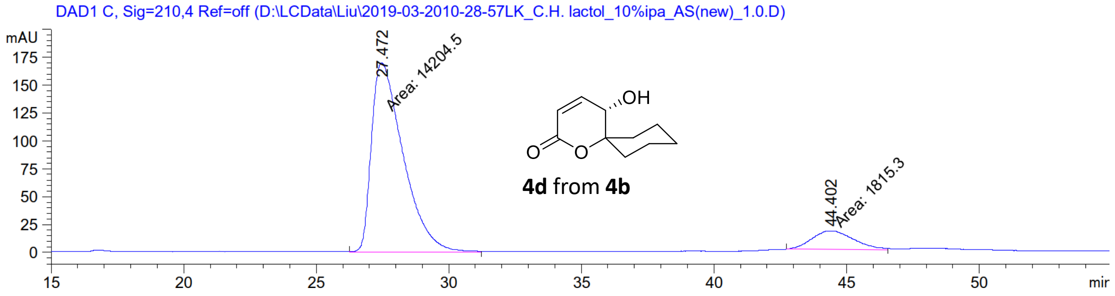

Supplement: Supplementary file 1 — Supporting Information [file CSSC-16-0-s002.pdf]
